# Responses of Processing Tomato Genotypes Under Varying NaCl Stress Levels and Durations

**DOI:** 10.3390/plants15101450

**Published:** 2026-05-09

**Authors:** Mingya Zhang, Qi Wang, Yudong Liu, Huiying Liu, Wei Xu, Xinting Yang, Shengqun Pang

**Affiliations:** 1 Department of Horticulture, College of Agriculture, Shihezi University, Shihezi 832003, China; zmia163@163.com (M.Z.); wangqi4@xjshzu.com (Q.W.); lyd-forever@163.com (Y.L.); hyliuok@aliyun.com (H.L.); xuwei0412@shzu.edu.cn (W.X.); 2 Laboratory of Cultivation Physiology and Germplasm Resources of Characteristic Fruits and Vegetables of Xinjiang Production and Construction Corps, Shihezi University, Shihezi 832003, China; 3 Information Technology Research Center, Beijing Academy of Agriculture and Forestry Sciences, Beijing 100097, China

**Keywords:** element content, photosynthetic characteristics, physiological indicators, salt stress

## Abstract

Currently, the escalating global problem of soil salinization severely limits the yield and quality of processing tomatoes. However, the differential responses and salt-tolerance strategies among processing tomato genotypes with different salt tolerances under salt stress remain largely elusive. Therefore, this study used salt-tolerant genotype ‘S39’ and salt-sensitive genotype ‘S37’ as materials. Seeds were sown in plug trays, and seedlings at the two-leaf-one-heart stage were transplanted into hydroponic containers filled with Hoagland nutrient solution. When seedlings reached the four-leaf-one-heart stage, they were exposed to NaCl treatments of 0 mM (control), 120 mM (Na120), and 180 mM (Na180). Plant samples were collected at 3, 6, and 9 days after treatment to determine growth parameters, physiological indices, and gene expression levels, aiming to reveal the dynamic differential responses to salt stress between the two processing tomato genotypes. The results demonstrated that the inhibitory effect of NaCl on the growth of processing tomatoes was aggravated with increasing NaCl concentration and treatment duration. The most significant difference in salt tolerance between the two genotypes was observed at 9 days under 180 mM NaCl treatment. At this sampling point, the relative salt-stress indices of superoxide dismutase (SOD) activity, peroxidase (POD) activity, soluble sugar content, proline content, chlorophyll a, chlorophyll b, and total chlorophyll (a + b) in ‘S39’ were significantly higher than those in ‘S37’ by 31.55%, 53.40%, 66.70%, 65.07%, 20.80%, 15.74%, and 19.44%, respectively. In addition, Na contents in roots and stems, as well as K contents in stems and leaves, were significantly higher in ‘S39’ than in ‘S37’ by 43.40%, 8.67%, 22.08%, and 21.99%, respectively. In contrast, relative electrolyte leakage and malondialdehyde (MDA) content in ‘S37’ were 15.54% and 12.44% higher than those in ‘S39’. In addition, photosynthetic parameters, including net photosynthetic rate (*A*_net_), stomatal conductance (*g*_s_), intercellular CO_2_ concentration (*C*_i_), transpiration rate (*E*), and chlorophyll fluorescence parameters, were more stable in ‘S39’ than in ‘S37’. In conclusion, ‘S39’ possesses stronger salt tolerance via a multi-level regulatory strategy involving an enhanced antioxidant enzyme system, elevated accumulation of osmoregulatory substances, improved mineral ion balance, and increased stability of the photosynthetic apparatus. This study provides a comprehensive multi-level analysis of the differential salt tolerance mechanisms in processing tomato genotypes with contrasting salt tolerances and lays a theoretical basis for the screening and identification of salt-tolerant germplasm in processing tomatoes.

## 1. Introduction

Under the dual negative effects of global climate change and human activities, soil salinization has gradually become one of the most critical factors threatening the stability of global ecosystems [1]. Soil salinization refers to the process in which soluble salt ions continuously accumulate in the surface layer of soil, thereby altering its physical and chemical properties, degrading its basic characteristics, and reducing overall soil quality [2]. It has become the most widespread form of soil degradation in arid and semi-arid regions [3]. Statistics indicate that more than one-tenth of the world’s land area is affected by salinization [4]. Moreover, factors such as global warming, improper irrigation and fertilization methods, and rising groundwater levels continue to exacerbate soil salinization, posing a serious challenge to sustainable agricultural development [5,6]. Plant salt tolerance varies by species. Generally, plants showing severe growth inhibition at 50–100 mM NaCl are defined as salt-sensitive plants; halophytes can tolerate NaCl concentrations exceeding 250 mM; and intermediate species capable of sustaining growth under moderate salinity are classified as moderately salt-tolerant plants [7]. The processing tomato (*Solanum lyacopersicum* L.) is a moderately salt-tolerant vegetable [8]. A relevant study has shown that the salt tolerance threshold of tomato is approximately 9.6 dS/m [9]. Beyond this threshold, salt stress significantly inhibits the growth and development, photosynthetic efficiency, and fruit quality of processing tomato, leading to a substantial decrease in yield [10].

Processing tomato, rich in lycopene, carotenoids, polyphenols, and other nutrients [11], serves as the main raw material for ketchup, tomato juice, canned tomatoes, and other processed products [12]. Regular consumption of processing tomato helps reduce the risk of cancer and cardiovascular diseases and protects eyesight [13]. Therefore, in-depth research on the tolerance mechanisms of processing tomato with regard to salt stress is of great significance for the exploration of strategies by which to improve salt tolerance and promote the sustainable development of the processing tomato industry.

In recent years, significant progress has been made in elucidating the mechanisms underlying plant salt tolerance, encompassing multiple aspects such as ion homeostasis [14], osmotic adjustment [15], and antioxidant enzyme systems [16]. Soil salinization leads to the accumulation of large amounts of Na^+^ in plants [17], resulting in imbalanced K^+^/Na^+^ and Na^+^/Ca^2+^ ratios that affect cell turgor and function, thereby limiting water and nutrient uptake [18]. This compromises the integrity of cell membranes, DNA, and proteins, and hinders the absorption of nutrients and water [19,20], leading to negative phenotypes such as reduced leaf area [21], leaf wilting, and shrinkage [22]. The regulation of ion homeostasis is one of the mechanisms by which plants respond to salt stress [23]. Plants can regulate salt tolerance by modulating the transport of different elements from roots to various above-ground organs, whereas excessively high salt concentrations directly impair this element transport capacity and cause severe damage to plants [24]. Salt-tolerant plants enhance salt tolerance by modulating ion channels [25] to restrict Na^+^ uptake and maintain a high K^+^/Na^+^ ratio [26]. High chlorophyll content and favorable ion homeostasis contribute to improved plant salt tolerance [27]. Salt stress disrupts the structure of photosynthetic reaction centers in chloroplasts and reduces photosynthetic parameters such as net photosynthetic rate, stomatal conductance, and intercellular CO_2_ concentration [28]. Meanwhile, chlorophyll content declines, leading to reduced photosynthetic efficiency [29]. This effect is particularly pronounced under high-concentration salt stress, which accelerates the degradation of photosynthetic pigments [30]. Chlorophyll fluorescence parameters serve as indicators when studying plant tolerance to abiotic stress [31]; related studies have shown that salt stress significantly decreases the maximum photochemical efficiency (F_v_/F_m_) and the actual quantum yield of photosystem II (Y(II)) by disrupting chloroplast structure and inhibiting the photosynthetic electron transport chain [32,33].

Enhancing antioxidant capacity is an effective strategy for improving plant salt tolerance [34]. Under salt stress, plants maintain reactive oxygen species balance via enzymatic and non-enzymatic antioxidant systems [35]; for instance, superoxide dismutase (SOD), peroxidase (POD), and ascorbate peroxidase (APX) play critical roles in defending against oxidative stress [36,37]. Studies have indicated that salt-tolerant genotypes exhibit stronger antioxidant capacity than salt-sensitive genotypes [38]. Moreover, several antioxidant-related genes have been cloned from crops such as rice [39], *Arabidopsis* [40], and soybean [41]. In addition, osmoprotectants, including proline, betaine, and mannitol, constitute key protective mechanisms in plants under salt stress [38,42]. Plants can also enhance salt stress tolerance through hormonal regulation involving abscisic acid, auxin, cytokinin, and other phytohormones [43,44]. In recent years, nanomaterials have exhibited promising potential for improving plant salt tolerance. For example, Ag and Si nanoparticles enhance plant salt tolerance by protecting photosynthesis, scavenging reactive oxygen species, and alleviating ion toxicity and osmotic stress [27,45,46].

Although numerous studies have investigated the salt tolerance mechanisms of tomato, most have focused on a single genotype or a limited set of physiological indicators. The differences in multi-indicator tolerance among germplasms with contrasting salt tolerance under various salt concentrations and stress durations remain to be further explored. In this study, to test the hypothesis that processing tomato germplasms with different salt tolerance genotypes employ distinct physiological and biochemical tolerance strategies under salt stress—strategies that may involve antioxidant enzyme activity, osmolyte accumulation, ion homeostasis, and photosynthetic characteristics—two processing tomato germplasms with contrasting salt tolerances were used as materials in a hydroponic experiment under different salt concentrations and stress durations. By measuring multiple indicators, including antioxidant enzyme activity, ion homeostasis, and photosynthetic characteristics, we conducted a multi-dimensional comparative analysis of the biochemical response differences between the two germplasms under various NaCl concentrations and stress durations. This study aims to elucidate the physiological and biochemical mechanisms underlying the response of processing tomatoes to saline stress, thereby providing a theoretical basis for the improvement of the utilization efficiency of salinized land, exploring effective strategies to cope with salt stress, and breeding new salt-tolerant varieties.

## 2. Materials and Methods

### 2.1. Experimental Materials

The experimental materials consisted of high-generation inbred tomato lines: the salt-tolerant line ‘S39’ and the salt-sensitive line ‘S37’, both provided by the Tomato Processing Research Group, College of Agriculture, Shihezi University Xinjiang, China. This classification was determined through phenotypic identification of 50 germplasm resources during the germination period [47] and seedling stage under salt stress.

### 2.2. Experimental Treatments and Sample Collection

**Figure 1:**
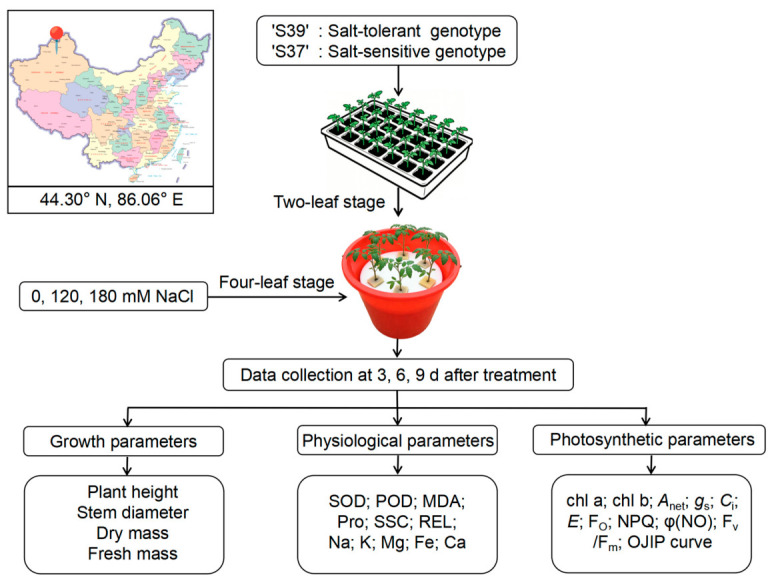
Schematic diagram of the experimental design. The experiment was conducted from 23 June 2024 to 5 August 2024 in a glass greenhouse at the College of Agriculture, Shihezi University (44.30° N, 86.06° E). The map was downloaded from https://bajiu.cn/ditu/?en2 (accessed on 23 April 2026). The upper left inset shows the location of the experimental site. Salt stress treatment was applied when the seedlings reached the four-leaf stage, using NaCl concentrations of 0 mM (control), 120 mM (Na120), and 180 mM (Na180). Samples were collected on days 3, 6, and 9 of NaCl treatment for the determination of relevant indicators. Abbreviations: SOD, superoxide dismutase; POD, peroxidase; MDA, malondialdehyde; Pro, proline; SSC, soluble sugar content; REC, relative electrical conductivity; Chl a, chlorophyll a; Chl b, chlorophyll b; *A*_net_, net photosynthetic rate; *E*, transpiration rate; *C*_i_, intercellular CO_2_ concentration; *g*_s_, stomatal conductance; F_0_, initial chlorophyll fluorescence; NPQ, non-photochemical quenching; φ(NO), quantum yield of non-regulated energy dissipation; F_v_/F_m_, maximum quantum yield of PSII photochemistry; OJIP curve, OJIP chlorophyll fluorescence transient.

The experiment was conducted from 23 June 2024 to 5 August 2024 in a glass greenhouse at the College of Agriculture, Shihezi University (44.30° N, 86.06° E). The photoperiod ranged from 12/12 h to 14/10 h (day/night), the temperature ranged from 27 to 35 °C, and the relative humidity ranged from 35% to 60%. Seeds of the two genotypes were germinated and then transferred to seedling trays filled with a mixture of vermiculite and peat at a ratio of 1:1 (*V*:*V*) (Figure 1). When the seedlings reached the two-leaf stage, uniform, representative and intact plants were selected and transplanted into hydroponic buckets containing 10 L of Hoagland nutrient solution. The buckets were 30 cm in both diameter and height, with six plants per bucket. A completely randomized block design was adopted in this experiment. To ensure sufficient sample availability, ten hydroponic buckets were arranged for each salt concentration treatment. When the seedlings reached the four-leaf stage, NaCl treatment was initiated at concentrations of 0 mM (control), 120 mM (Na120), and 180 mM (Na180), which were determined based on preliminary screening results from our research group [48]. The electrical conductivity of the Hoagland nutrient solution containing 0, 120, and 180 mM NaCl was measured to be approximately 2.40, 15.00, and 20.20 dS/m, respectively. The nutrient solution was renewed every 3–5 days. On days 3, 6, and 9 after salt stress, growth parameters were measured, and samples were collected for physiological and biochemical indicators and gene expression analyses. The third and fourth fully expanded leaves from the top were used for sampling.

### 2.3. Measurement of Growth Indicators

On days 3, 6, and 9 following salt stress, growth parameters were measured at the same time each day (Figure 1), with three biological replicates per treatment. Three uniform growth, representative plants free of mechanical damage were selected from each treatment and marked for repeated measurements of plant height and stem diameter. Plant height (cm) was measured from the cotyledonary node to the apical meristem using a measuring tape. Stem diameter (mm) was determined at the cotyledonary node using a 0–150 mm digital caliper with 0.01 mm accuracy (DELIXI, Yueqing, Zhejiang, China). Three additional plants were selected for destructive sampling to determine fresh and dry mass. Seedlings were harvested from hydroponic buckets, rinsed with distilled water, and blotted dry with filter paper to remove excess surface moisture. Fresh mass (g) was measured using an SQP electronic analytical balance (Sartorius, Beijing, China). Samples were then placed in paper bags and oven-dried at 105 °C for 10 min to inactivate enzymes, followed by drying at 80 °C until constant mass was attained (mass difference < 0.01 g between two consecutive measurements). After cooling to room temperature, the samples were weighed to obtain plant dry mass (g).

### 2.4. Physiological and Biochemical Indicators

Enzyme extraction: A 0.5 g sample was mixed with 5 mL of 0.05 M phosphate buffer solution (PBS, pH 7.8) and ground to a homogenate in an ice bath. The homogenate was centrifuged at 13,000 rpm and 4 °C for 20 min, and the supernatant was used as the crude extract for assaying superoxide dismutase (SOD) and peroxidase (POD) activities.

All absorbance values were measured using a UV2600 spectrophotometer (Shimadzu, Suzhou, Jiangsu, China). The centrifugation step was performed using a Heraeus 8R high-speed refrigerated centrifuge (Thermo Fisher Scientific, Waltham, MA, USA).

Superoxide dismutase (SOD) activity was determined according to the nitroblue tetrazolium (NBT) method [49]. Briefly, 50 μL of enzyme extract and 3 mL of NBT reaction solution were added to a transparent test tube. In the control tube, the enzyme extract was replaced with PBS (pH 7.8). After mixing, one control tube was placed in the dark, while the other tubes were incubated under 4000 lx light for 20 min.

The control tube kept in the dark was used as a blank for zero adjustment. The absorbance was measured at 560 nm and the SOD activity was calculated as follows (Equation (1)):

(1) $$\mathrm{SOD}\;\mathrm{activity}\;({U\;g}^{-1}\;\mathrm{FM}\cdot h^{-1})=\frac{(A_{0}-A_{S})\;V_{t}}{{0.5A}_{0}\;FM\;V_{S}\;t}$$

where *A*_0_: absorbance of the control tube under light; *As*: absorbance of the sample tube under light; *Vt*: total volume of the enzyme extract (mL); *Vs*: volume of crude enzyme extract used in the assay (mL); *t*: reaction time (h); and *FM*: fresh mass of the sample (g).

Peroxidase (POD) activity was determined according to the guaiacol method [49]. In a cuvette, 1.7 mL of PBS (pH 7.0), 100 μL of 1% guaiacol, 100 μL of 20 mM H_2_O_2_, and 100 μL of enzyme extract were added sequentially and mixed. A control cuvette containing 0.05 M PBS instead of the enzyme extract was used as a blank for zero adjustment. The absorbance was measured at 470 nm every 20 s for a total of 1 min (Equation (2)):

(2) $$\mathrm{POD}\;\mathrm{activity}\;({U\;g}^{-1}\;\mathrm{FM}\;h^{-1})\;=\frac{\Delta A470\;V_{t}}{\;0.01FM\;V_{S}\;t}$$

where Δ*A*470: change in absorbance over the reaction time; *FM*: fresh mass of the sample (g); *t*: reaction time (h); *Vt*: total volume of the enzyme extract (mL); and *Vs*: volume of crude enzyme extract used in the assay (mL).

Malondialdehyde (MDA) content was determined according to the thiobarbituric acid (TBA) method [50]. A 0.2 g sample was placed in a mortar with a small amount of quartz sand and 2 mL of 5% trichloroacetic acid (TCA), and ground to a homogenate. The homogenate was filtered into a centrifuge tube and the volume was adjusted to 5 mL with distilled water. The mixture was centrifuged at 4000 r/min for 10 min. A 2 mL aliquot of the supernatant was transferred to a test tube and mixed with 2 mL of TBA solution. The mixture was heated in a boiling water bath for 15 min, then centrifuged at 4000 r/min for 10 min. The absorbance of the supernatant was measured at 450, 532, and 600 nm, and the MDA content was calculated using Equation (3):

(3) $$\mathrm{MDA}\;\mathrm{content}\;(\mathrm{mmol}\;g^{-1}\;\mathrm{FM})\;=\;[6.452\;(A652\;-\;A600)\;-\;0.559\;A450]\frac{V_{t}}{V_{s\;FM}}$$

where *A*652: absorbance at 652 nm; *A*600: absorbance at 600 nm; *Vt*: total volume of the enzyme extract (mL); *Vs*: volume of extract used in the assay (mL); and *FM*: fresh mass of the sample (g).

Proline content was determined according to the acid ninhydrin method [51]. Briefly, 0.5 g of sample was extracted with 10 mL of 3% sulfosalicylic acid in a boiling water bath for 30 min. After cooling and filtration, 2 mL of the filtrate was mixed with 2 mL of glacial acetic acid and 3 mL of acid ninhydrin reagent in a 25 mL stoppered test tube, and heated in a boiling water bath for 40 min. After cooling, 5 mL of toluene was added, and the mixture was shaken for 30 s. The upper red layer was collected after phase separation, and the absorbance was measured at 520 nm against a blank to calculate proline content (Equation (4)):

(4) $$\mathrm{proline}\;\mathrm{content}\;(\mu g\;g^{-1}\;\mathrm{FM})\;=\;\frac{\;5C}{FM}$$

where *C*: proline content of the sample obtained from the standard curve (μg); *FM*: fresh mass of the sample (g); and 5: ratio of the total extraction volume (10 mL) to the aliquot volume used for measurement (2 mL).

Soluble sugar content was determined according to the anthrone colorimetric method [52]. A 0.5 g sample was placed in a 25 mL stoppered test tube containing 5 mL of distilled water and heated in a boiling water bath HH-8 (LICHEN, Shaoxing, Zhejiang, China) for 30 min. The extract was filtered into a 25 mL volumetric flask. The leaf residue was retained in the stoppered test tube, and 5 mL of distilled water was added for a second extraction in a boiling water bath for 30 min. After cooling, the extract was filtered into the same volumetric flask, and the volume was adjusted to 25 mL with distilled water. A 0.5 mL aliquot of the extract was transferred to a stoppered test tube, mixed with 1.5 mL of distilled water, 0.5 mL of anthrone reagent, and 5 mL of concentrated sulfuric acid, and then heated in a boiling water bath for 1 min. The absorbance was measured at 630 nm using a blank as reference, and the soluble sugar content was calculated using Equation (5):

(5) $$\mathrm{soluble}\;\mathrm{sugar}\;\mathrm{content}\;(\mu g\;g^{-1}\;\mathrm{FM})\;=\;\frac{m\;Vt\;D}{10^{6}FM\;Vs}$$

where *m*: soluble sugar content of the sample obtained from the standard curve (μg); *Vt*: total volume of the enzyme extract (mL); *Vs*: volume of extract used in the assay (mL); *D*: dilution factor; and *FM*: fresh mass of the sample (g).

Leaf relative electrical conductivity was determined using a DDSJ-307F conductivity meter (Leici, Shanghai, China). The fourth leaf from the top was cut into small rectangular pieces, and 0.2 g of the leaf samples was placed in a 25 mL stoppered test tube containing 10 mL of ultrapure water. After standing for 12 h, the initial electrical conductivity (EC_1_) was recorded. The samples were then heated in a boiling water bath for 30 min, and the final electrical conductivity (EC_2_) was measured. The relative electrical conductivity (REC) was calculated using Equation (6):

(6) $$\mathrm{REC}(\%)\;=\;EC1\;E{C2}^{-1}\times \;100\%$$

### 2.5. Determination of Elemental Content

For each treatment, three plants were taken, washed with distilled water, and then the roots, stems, and leaves were separated and placed in an oven at 105 °C for 10 min to inactivate enzymes. After that, the oven temperature was adjusted to 80 °C and the samples were dried to constant mass. The samples were digested using the HNO_3_-H_2_O_2_ method, and the contents of Na, K, Mg, Ca, and Fe were determined using an inductively coupled plasma emission spectrometer iCAP PRO X (Thermo Fisher Scientific, Bremen, Germany). The K/Na ratio in roots, stems, and leaves was calculated. Furthermore, the absorption and distribution of different nutrient elements in processing tomato were compared by calculating the transport coefficients [24] of the above elements from root to stem and from root to leaf, as follows (Equation (7)):

(7) $$S_{X,\mathrm{Na}}\;=\;\frac{Y[X{Na}^{-1}]}{Root[X{Na}^{-1}]}$$

where *X* represents the elements K, Mg, Ca, and Fe, and *Y* represents the stem or leaf. The larger the ratio of S_X,Na_, the stronger the ability of the root to control Na and promote the transport of element X to Y.

### 2.6. Leaf Photosynthetic Pigments and Photosynthetic Parameters

Chlorophyll content was extracted using the 95% ethanol extraction method [53] at a ratio of 1:1 (*V*:*V*). For each treatment, 0.1 g of fresh leaves were cut into small pieces, placed into stoppered test tubes containing 5 mL of 95% ethanol (*V*:*V*), and then kept in the dark for chlorophyll extraction (48 h), with three replicates. After the leaves became completely bleached, the absorbance of the extract was measured at 665 and 649 nm using a UV2600 spectrophotometer (Shimadzu, Suzhou, Jiangsu, China), and the chlorophyll content was calculated using Equations (8) and (9), as follows:

(8) $$Chl\;a\;content\;(\mathrm{mg}\;g^{-1}\;\mathrm{FM})=0.001(13.95OD665-6.88OD649)\;V\;{FM}^{-1}$$

(9) $$Chl\;b\;content\;(\mathrm{mg}\;g^{-1}\;\mathrm{FM})=0.001(24.96OD649-7.32OD665)\;V\;{FM}^{-1}$$

where *Chl a*: Chlorophyll a; *Chl b*: Chlorophyll b; *V*: volume of extract used in the assay (mL); and *FM*: fresh mass of the sample (g).

Photosynthetic parameters were measured between 10:00 a.m. and 12:00 p.m. on the fourth leaf from the top (counting downwards), with three plants measured per treatment, using an LI-6800 portable photosynthesis system (LI-COR, Lincoln, NE, USA) equipped with a red-blue LED light source (LI-6800-02), the light spectrum consisted of 90% red light and 10% blue light. The measured parameters included net photosynthetic rate (*A*_net_), stomatal conductance (*g*_s_), intercellular CO_2_ concentration (*C*_i_), and transpiration rate (*E*). The leaf chamber conditions were set as follows: photosynthetically active radiation at 1000 μmol m^−2^ s^−1^, CO_2_ concentration at 400 μmol mol^−1^, airflow rate at 500 μmol s^−1^, relative humidity at 50%, and temperature at 25 °C.

### 2.7. Measurement of Fast Chlorophyll Fluorescence Kinetics Curve and Chlorophyll Fluorescence Parameters

After transferring the plants from each salt stress treatment from the glass greenhouse to a dark room for 30 min of dark adaptation, three plants per treatment were selected, and the OJIP chlorophyll fluorescence transient curves of the fourth leaf from the top of processing tomato plants were measured using a multi-function plant efficiency analyzer (M-PEA, Hansatech Instruments, Norfolk, UK). The equations and definitions of the OJIP curves and JIP-test parameters are shown in Supplementary Table S1. Mo, initial slope of the OJIP fluorescence induction curve (off rate of RCs); V_I_, variable fluorescence at point I; V_J_, relative variable fluorescence at point J; φPo, the maximum quantum yield of primary PSII photochemistry; S_m_, area between the normalized OJIP; N, the number of Q_A_ redox turnovers before reaching Fm; Ψ_O,_ the probability with which a PSII-trapped electron is transferred from Q_A_ to Q_B_ in the electron transport chain; φE_O_, the quantum yield of electron transport flux from Q_A_ to Q_B_; ABS/RC, light energy absorbed per reaction center; TR_O_/RC, energy captured per reaction center for reduction of Q_A_ (at t = F_O_); ET_O_/RC, energy captured per reaction center for electron transfer (at t = F_O_); RC/CS_m_, number of active reaction centers per unit area (at t = F_m_); ABS/CS_m_, light energy absorbed per unit area (at t = F_m_); TR_O_/CS_m_, light energy captured per unit area (at t = F_m_); ET_O_/CS_m,_ quantum yield per unit area for electron transfer (at t = F_m_); DI_O_/CS_m_, heat dissipation per unit area (at t = F_m_); PI_abs,_ performance index on absorption basis; and PI_csm_, performance index on cross section basis. Chlorophyll fluorescence parameters were measured with an M-Imaging PAM-modulated chlorophyll fluorescence imaging system (WALZ, Effeltrich, Bavaria, Germany), including the initial fluorescence of photosystem II (Fo), maximum photochemical efficiency (F_v_/F_m_), quantum yield of non-regulated energy dissipation [φ(NO)], and non-photochemical quenching coefficient (NPQ). During the measurement of the OJIP curves and chlorophyll fluorescence parameters, the dark environment was maintained to avoid light interference with the fluorescence signals.

### 2.8. Quantitative Gene Expression Analysis by Quantitative Real-Time Reverse Transcription Polymerase Chain Reaction

Total RNA was extracted from the leaves of processing tomato plants using a plant RNA extraction kit RNP451-02 (Kediyuan, Shihezi, Xinjiang, China); cDNA was synthesized using a reverse transcription kit R202-02 (Xinbei Biotechnology, Shanghai, China), and qRT-PCR was conducted using a fluorescence quantitative PCR kit (Q204-01, Xinbei Biotechnology, Shanghai, China). The relative expression levels were calculated using the 2^–ΔΔCt^ method with *Actin* (microfilament cytoskeletal protein gene) as the internal reference gene. Among these were *SlSOD1* (encoding superoxide dismutase, directly related to SOD activity); *SlPOD1* (encoding peroxidase, directly related to POD activity); *SlTPP3* (encoding trehalose-6-phosphate phosphatase, a key enzyme in soluble sugar metabolism); and *SlP5CS* (encoding Δ^1^-pyrroline-5-carboxylate synthetase, the rate-limiting enzyme in proline biosynthesis). The primer sequences are indicated in Table 1.

### 2.9. Data Processing and Statistical Analysis

Relative salt-stress indices are widely used in the evaluation of plant salt tolerance [54,55]. To eliminate the inherent baseline differences between germplasms and facilitate comparison between the two genotypes, the activity of antioxidant enzymes, the content of osmotic regulating substances, electrical conductivity, and MDA content were converted into salt stress relative indices. This involved calculating the ratio of the values of these indicators under NaCl treatment to those under control treatment. The test data were organized, the mean ± standard deviation was calculated using Excel, and the plots were generated using Origin 2024. One-way analysis of variance (ANOVA) was performed using SPSS 25 software, and mean comparisons were conducted using Duncan’s multiple range test. A *p*-value < 0.05 was considered statistically significant. Furthermore, to explore the associations among major physiological indices under NaCl treatment, Spearman correlation analyses were performed separately for the two concentrations (Na120 and Na180) based on data simply normalized to the control (*p* < 0.05).

## 3. Results

### 3.1. Effects of NaCl Treatment on Growth Parameters of Processing Tomatoes

**Figure 2:**
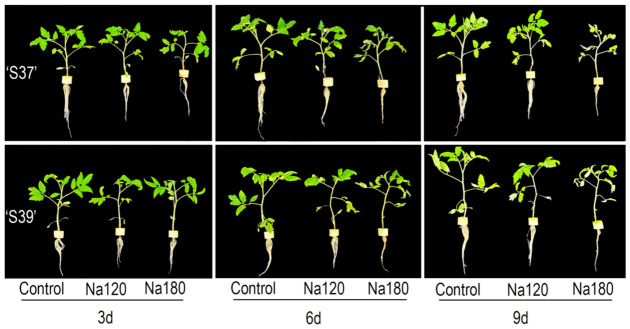
Morphological tolerance of processing tomato seedlings under NaCl stress. Note: Control, Na120, and Na180 represent Hoagland nutrient solution treatments containing 0, 120, and 180 mM NaCl, respectively. Treatment durations were 3, 6, and 9 days. ‘S37’ and ‘S39’ represent salt-sensitive and salt-tolerant processing tomato genotypes, respectively.

NaCl treatment inhibited the growth of the two genotypes (Table 2 and Figure 2). Leaf wilting occurred in ‘S37’ on day 9 under the Na120 treatment and on days 6 and 9 under the Na180 treatment, while leaf wilting in ‘S39’ occurred only on day 9 under the Na180 treatment. Although the NaCl treatment at different concentrations did not completely inhibit the growth of the two genotypes, the degree of inhibition increased with higher salt concentration and treatment time. At 6 and 9 days after NaCl treatment, the dry mass of ‘S37’ was significantly reduced compared with the control, while ‘S39’ showed no significant difference from the control. Additionally, NaCl treatment had the most potent inhibitory effect on fresh mass. Compared with the control, the fresh mass of ‘S37’ significantly decreased by 41.90%, 48.32%, and 52.02% on days 3, 6, and 9 under the Na120 treatment, respectively. For ‘S39’, fresh mass decreased by 20.61% on day 3 under the Na120 treatment, which was not significantly different from the control, but exhibited significant reductions of 39.73% and 50.95% on days 6 and 9, respectively. Compared with the control, the fresh mass of ‘S37’ under Na180 treatment decreased significantly by 44.05%, 58.59%, and 75.62% at 3, 6, and 9 days, while that of ‘S39’ decreased by 37.87%, 47.05%, and 69.52%, respectively. Under NaCl treatment, the decrease amplitude of fresh mass in the two genotypes was significantly higher than that of plant height, stem diameter and dry mass. This indicates that water imbalance was the primary manifestation of salt injury and that fresh mass could be used as an indicator for evaluating the salt tolerance of processing tomatoes.

### 3.2. Effects of NaCl Treatment on the Physiological Characteristics of Processing Tomatoes

**Figure 3:**
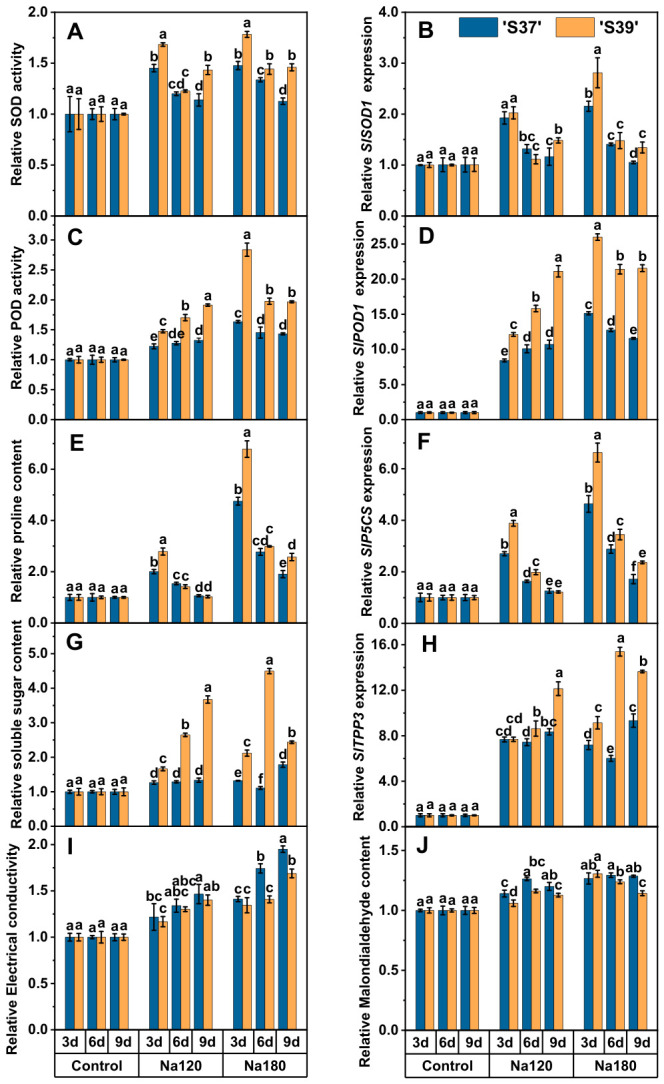
Effects of NaCl stress on physiological and biochemical parameters and gene expression in processing tomato seedlings. Note: Control, Na120, and Na180 represent Hoagland nutrient solution treatments containing 0, 120, and 180 mM NaCl, respectively, with treatment durations of 3, 6, and 9 days. ‘S37’ and ‘S39’ represent salt-sensitive and salt-tolerant processing tomato genotypes, respectively. (**A**): Superoxide dismutase (SOD) activity (U g^−1^ FM h^−1^); (**C**): Peroxidase (POD) activity [U g^−1^ FM h^−1^]; (**E**): Proline (Pro) content (μg g^−1^ FM); (**G**): Soluble sugar content (μg g^−1^ FM); (**I**): Relative electrical conductivity of leaves (%); (**J**): Malondialdehyde (MDA) content (mmol g^−1^ FM); (**B**,**D**,**F**,**H**): Relative expression levels of *SlSOD1*, *SlPOD1*, *SlTPP3*, and *SlP5CS*, respectively. Each treatment had three biological replicates. Statistical significance was determined by one-way ANOVA followed by Duncan’s multiple. A *p*-value < 0.05 was considered statistically significant, and significant differences are indicated by different letters.

The SOD activity, POD activity, and gene expression levels in ‘S37’ and ‘S39’ were higher under NaCl treatment than in the control (Figure 3A–D). Under Na120 treatment, the SOD activity and *SlSOD1* expression level in ‘S39’ first decreased and then increased with the extension of treatment time (Figure 3A,B). Under other treatments, the SOD activity and *SlSOD1* expression levels of ‘S37’ and ‘S39’ gradually decreased with the extension of treatment time. On day 6 of Na120 treatment, there was non-significant difference in SOD activity between ‘S37’ and ‘S39’. Similarly, on days 3 and 6 of Na120 treatment and day 6 of Na180 treatment, there was non-significant difference in *SlSOD1* expression between ‘S37’ and ‘S39’. Under the other treatments, both SOD activity and its gene expression level were significantly higher in ‘S39’ than in ‘S37’, suggesting that ‘S39’ possessed a stronger reactive oxygen species scavenging capacity. The changing trends of POD activity and *SlPOD1* expression in ‘S37’ and ‘S39’ were similar under NaCl treatment (Figure 3C,D). Under Na120 treatment, both POD activity and *SlPOD1* expression demonstrated a gradual increase with the extension of treatment time. Under Na180 treatment, however, they exhibited a gradual decreasing trend with increasing treatment time. This phenomenon may be attributed to long-term exposure to high NaCl concentrations, which caused cellular metabolic disturbance or enzyme protein denaturation and thereby inhibited the function of the antioxidant system. Nevertheless, ‘S39’ maintained significantly higher POD activity than ‘S37’. Meanwhile, the overall POD activity and *SlPOD1* expression level increased with the increase in NaCl concentration, indicating that high NaCl concentrations can continuously activate the function of the antioxidant system. Under all treatments, ‘S39’ exhibited significantly higher POD activity and corresponding gene expression than ‘S37’, indicating its stronger ROS scavenging capacity at high salt concentrations.

The proline content, soluble sugar content, and the expression levels of related genes of ‘S37’ and ‘S39’ under NaCl treatment are all higher than those of the control (Figure 3E–H). On days 6 and 9 of Na120 treatment and day 6 of Na180 treatment, there was non-significant difference in the proline content between ‘S37’ and ‘S39’ (Figure 3E). On day 3 of Na120 treatment, there was non-significant difference in the expression levels of *SlTPP3* between ‘S37’ and ‘S39’ (Figure 3H). On day 9 of Na120 treatment, there was non-significant difference in the expression levels of *SlP5CS* between ‘S37’ and ‘S39’ (Figure 3F). Under other treatments, the contents of proline and soluble sugar (Figure 3G), along with the expression levels of their related genes, were significantly higher in ‘S39’ than in ‘S37’, indicating that ‘S39’ may have a stronger osmotic regulatory ability. Under Na120 treatment, the soluble sugar content of ‘S39’ and the expression level of *SlTPP3* indicated a significant upward trend. Under Na180 treatment, the soluble sugar content of ‘S37’ and the expression level of the *SlTPP3* first decreased and then increased, while ‘S39’ indicated a trend of first increasing and then decreasing, indicating that the soluble sugar content of ‘S39’ and its related genes responded more rapidly to NaCl treatment. The proline content of ‘S37’ and ‘S39’ and the expression level of *SlP5CS* both indicated a gradual decrease with the delay of treatment time, suggesting that the expression of proline synthesis-related genes was inhibited as treatment time increased, leading to a reduction in proline accumulation.

The relative conductivity of ‘S37’ and ‘S39’ increased with higher NaCl concentrations and longer treatment durations (Figure 3I). Under NaCl treatment, significant differences between the two lines were observed only on days 6 and 9 under the Na180 treatment, during which ‘S39’ exhibited significantly lower relative conductivity than ‘S37’. Non-significant differences were observed under other treatment conditions. Specifically, compared with the control, the relative conductivity of ‘S37’ increased by 74.25% and 95.10% on days 6 and 9 of Na180 treatment, whhile that of ‘S39’ increased by 40.81% and 68.86%, respectively.

The relative MDA content of ‘S37’ and ‘S39’ both increased under NaCl treatment and tended to rise with higher NaCl concentration at the same timepoints (Figure 3J). Under Na120 treatment, both lines indicated a trend of first increasing and then decreasing with prolonged exposure. The maximum increase occurred on day 6, with MDA levels rising by 26.29% in ‘S37’ and 16.20% in ‘S39’. At this stage, the MDA content of ‘S39’ was significantly lower than that of ‘S37’. Under Na180 treatment, when compared with the control, ‘S39’ exhibited the highest MDA increase on day 3 (30.61%), while ‘S37’ peaked on day 6 (29.43%). With the exception of day 3, when a non-significant difference was observed between the two lines, the MDA content of ‘S39’ remained significantly lower than that of ‘S37’ on days 6 and 9.

### 3.3. Effect of NaCl Treatment on Element Content in Different Tissues of Processing Tomatoes

**Figure 4:**
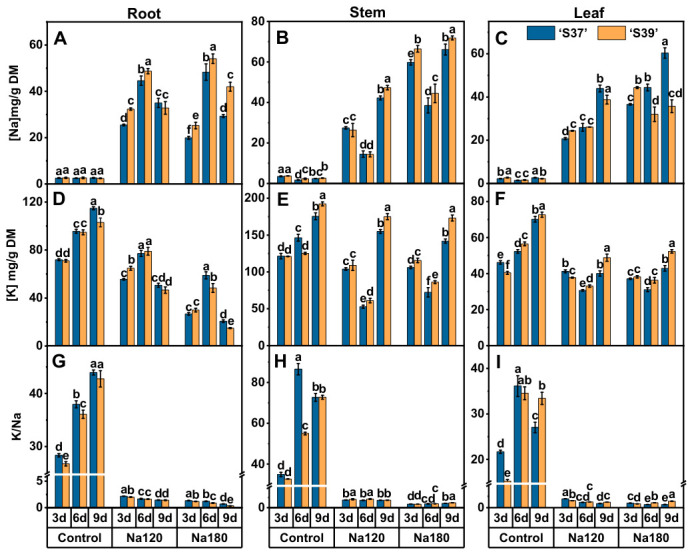
The element content in the roots, stems, and leaves of tomato under different NaCl concentrations. Note: Control, Na120, and Na180 represent Hoagland nutrient solution treatments containing 0, 120, and 180 mM NaCl, respectively. The treatment durations were 3, 6, and 9 days. ‘S37’ and ‘S39’ represent salt-sensitive and salt-tolerant processing tomato genotypes, respectively. Elemental contents in roots, stems, and leaves were measured for each genotype at each treatment duration. Na content in roots (**A**), stems (**B**), and leaves (**C**). K content in roots (**D**), stems (**E**), and leaves (**F**), Na/K content in roots (**G**), stems (**H**), and leaves (**I**). Each treatment had three biological replicates. Statistical significance was determined by one-way ANOVA followed by Duncan’s multiple. A *p*-value < 0.05 was considered statistically significant, and significant differences are indicated by different letters.

Under the control treatment, the Na content in the two genotypes was uniformly distributed in the roots, stems, and leaves (Figure 4A–C). As the NaCl treatment concentration or time increased, the Na content in the roots, stems, and leaves of ‘S37’ and ‘S39’ exhibited a gradual increase overall. Under NaCl treatment, the overall trend indicated that the Na accumulated by ‘S39’ in the roots and stems was more than that of ‘S37’ and that the Na accumulated by ‘S37’ in the leaves was more than that of ‘S39’. In the roots, the increase in Na was greatest on day 6 of NaCl treatment when compared with the control. Under Na120 treatment, ‘S37’ and ‘S39’ increased by 15.94 and 17.05 times, respectively. Under Na180 treatment, these increased by 17.82 and 18.19 times, respectively. In the stems, the increase in Na content was greatest on day 9 of NaCl treatment when compared with the control. Under Na120 treatment, ‘S37’ and ‘S39’ increased by 16.47 and 16.88 times, respectively. Under Na180 treatment, these increased by 26.37 and 26.18 times, respectively.

The K content in the roots, stems, and leaves of the two genotypes gradually increased over time under the control treatment (Figure 4D–F). Under the NaCl treatment, as the treatment time extended, the K content in the roots initially increased and then decreased, while in the stems and leaves, it initially decreased and then increased. The K accumulated in the roots of ‘S37’ was more than that in ‘S39’, and the K content in the stems and leaves of ‘S39’ was also higher than that of ‘S37’. Under Na120 treatment, the K content in the stems of both genotypes was highest on days 3 and 9, while the K content in the roots was highest on day 6. Under Na180 treatment, the K content in the stems was highest in both genotypes.

The K/Na ratio in the roots, stems, and leaves of the two genotypes significantly decreased when compared with the control under NaCl treatment (Figure 4G–I). In the root, there was non-significant difference in K/Na between ‘S37’ and ‘S39’ on day 9 of the control treatment and days 6 and 9 of the Na120 treatment. Under the other treatments, ‘S39’ was significantly lower than ‘S37’. In the stems, there was non-significant difference between ‘S37’ and ‘S39’ on days 3 and 6 of control treatment, day 9 of Na120 treatment, and days 3 and 6 of Na180 treatment. However, on day 6 of Na120 treatment and day 9 of Na180 treatment, ‘S39’ was significantly higher than ‘S37’. In the leaves, under the control treatment, ‘S37’ was significantly higher than ‘S39’ on day 3 of Na120 and Na180 treatments, while there was a non-significant difference between ‘S37’ and ‘S39’ on day 6 of the control and Na120 treatments. On day 6 of Na120 treatment and day 9 of each treatment, ‘S39’ was significantly higher than ‘S37’.

In summary, under NaCl treatment, ‘S39’ can sense the NaCl stress signal more quickly and activate the ion transport system to reduce Na accumulation when compared with ‘S37’. This maintains a higher K/Na ratio in the above-ground part over a long period of NaCl treatment, demonstrating better salt tolerance.

### 3.4. Effect of NaCl Treatment on the Selective Transport Capacity of Nutrient Elements in Different Parts of Processing Tomatoes

With increasing NaCl concentration, the ability of the two genotypes to control Na and promote the transport of K, Mg, Ca, and Fe to the above-ground part generally showed a trend of first increasing and then decreasing (Table 3). Specifically, on day 3 of NaCl treatment, the root–leaf S_Mg,Na_ and S_Fe,Na_ of ‘S37’ gradually decreased, and on day 6, the root–stem S_K,Na_ and the root–leaf S_K,Na_, S_Mg,Na_, and S_Fe,Na_ also decreased. On days 6 and 9 of NaCl treatment, the S_Mg,Na_ of ‘S39’ gradually increased. On day 9, the root–stem and root–leaf S_K,Na_ of both genotypes gradually increased, with a greater increase in ‘S39’ than in ‘S37’. Except for S_K,Na_, the overall values of S_Mg,Na_, S_Ca,Na_, and S_Fe,Na_ followed the pattern of root–leaf > root–stem, indicating that, with the exception of K, roots control Na^+^ and promote the transport of Mg, Ca, and Fe to leaves more strongly than to stems. On day 3 of NaCl treatment, the increases in root–leaf S_K,Na_, S_Ca,Na_, and S_Fe,Na_ relative to the control were all higher in ‘S39’ than in ‘S37’. Under the Na120 treatment, the increases in root–stem S_Mg,Na_ and S_Ca,Na_ relative to the control were also greater in ‘S39’ than in ‘S37’. With the exception of root–stem S_Ca,Na_ on day 6 of Na180 treatment and root–stem S_K,Na_ and S_Ca,Na_ as well as root–leaf S_Ca,Na_ on day 9 of Na120 treatment, the increases in S_K,Na_, S_Mg,Na_, S_Ca,Na_, and S_Fe,Na_ under all other treatments relative to the control were higher in ‘S39’ than in ‘S37’. In conclusion, under NaCl treatment, ‘S39’ possesses a stronger ability than ‘S37’ to preferentially transport mineral elements to the above-ground parts.

### 3.5. Effects of NaCl Treatment on Leaf Photosynthetic Pigments in Processing Tomatoes

**Figure 5:**
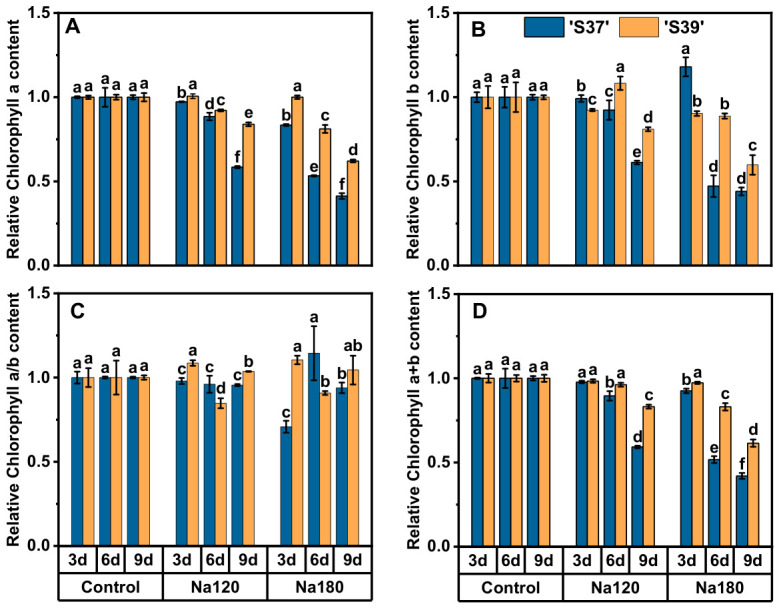
Relative chlorophyll content (a, b, a/b, a + b) (**A**–**D**) in processing tomato leaves: NaCl and temporal effects. Note: Control, Na120, and Na180 represent Hoagland nutrient solution treatments containing 0, 120, and 180 mM NaCl, respectively. The treatment durations were 3, 6, and 9 days. ‘S37’ and ‘S39’ represent salt-sensitive and salt-tolerant processing tomato genotypes, respectively. Each treatment had three biological replicates. Statistical significance was determined by one-way ANOVA followed by Duncan’s multiple. A *p*-value < 0.05 was considered statistically significant, and significant differences are indicated by different letters.

‘S37’ and ‘S39’ showed significant differences in the tolerance of chlorophyll content-related indicators to NaCl treatment (Figure 5). Under Na120 treatment, except for the Chl b content of ‘S39’ (Figure 5B), which showed an initial increase followed by a decrease, the Chl a (Figure 5A), Chl b, and total Chl a + b (Figure 5D) of both genotypes exhibited a gradual declining trend with prolonged treatment time, with the decline in ‘S37’ being significantly greater than that in ‘S39’. Specifically, by day 9, the Chl a, Chl b, and total Chl a + b of ‘S37’ decreased by 41.53%, 38.78%, and 40.80%, respectively, when compared with the control. In contrast, ‘S39’ showed reductions of 16.09%, 19.00%, and 16.89% in Chl a, Chl b, and total Chl a + b, respectively, by day 9. Regarding the Chl a/b ratio (Figure 5C), no significant changes were observed in ‘S37’ with prolonged Na120 treatment. For ‘S39’, after a brief decline from day 3 to day 6, the ratio recovered to 103.61% of the control by day 9. ‘S39’ was significantly lower than ‘S37’ on day 6 but significantly higher than ‘S37’ on days 3 and 9, indicating its stronger photoprotective mechanism.

Na180 treatment significantly inhibited chlorophyll synthesis or accelerated chlorophyll degradation in both genotypes. By day 6, the Chl a, Chl b, and total Chl a + b of ‘S37’ decreased by 46.68%, 52.81%, and 48.32%, respectively, when compared with the control. For ‘S39’, the reductions were 18.84%, 11.17%, and 16.96%, respectively. By day 9, these indicators further declined significantly in both genotypes. Overall, under Na180 treatment, the chlorophyll content-related indicators were higher in ‘S39’ than in ‘S37’. The total Chl a + b of ‘S37’ showed a brief increase from day 3 to day 6 but significantly decreased by 6.12% compared with the control by day 9. In contrast, ‘S39’ exhibited a temporary reduction from day 3 to day 6, followed by a recovery to 104.46% of the control by day 9. In summary, under the same NaCl treatment, ‘S37’ suffered more severe damage to its photosynthetic system, while ‘S39’ demonstrated stronger NaCl adaptation and recovery capabilities.

### 3.6. Effects of NaCl Treatment on Leaf Photosynthetic Parameters in Processing Tomatoes

With increasing NaCl concentration and treatment duration, the *A*_net_, *E*, and *g*_s_ of both genotypes showed a decreasing trend, with the most pronounced decline observed on day 9 of the NaCl stress (Table 4). On day 9 of the Na120 treatment, the *A*_net_, *E*, and *g*_s_ of ‘S37’ were significantly lower than those of the control by 31.31%, 40.74%, and 54.22%, respectively. For ‘S39’, *E* and *g*_s_ were significantly lower than those of the control by 21.27% and 41.84%, respectively, whereas *A*_net_ and *C*_i_ showed no significant difference relative to the control. On day 9 of Na180 treatment, the *A*_net_, *E*, and *g*_s_ of ‘S37’ and ‘S39’ were 73.20%, 80.72%, 81.16% and 64.74%, 63.05%, 69.94% lower than the control, respectively. Under the same NaCl treatment conditions, the decline in ‘S37’ was greater than that in ‘S39’. The trends in *C*_i_ differed between the two genotypes. Under Na120 treatment, both genotypes showed a significant decrease when compared with the control on days 3 and 6, with no significant difference from the control on day 9. Under Na180 treatment, *C*_i_ in ‘S37’ increased substantially on days 6 and 9, while no significant difference was observed in ‘S39’ compared with the control.

### 3.7. Effects of NaCl Treatment on Chlorophyll Fluorescence Parameters in Processing Tomatoes

**Figure 6:**
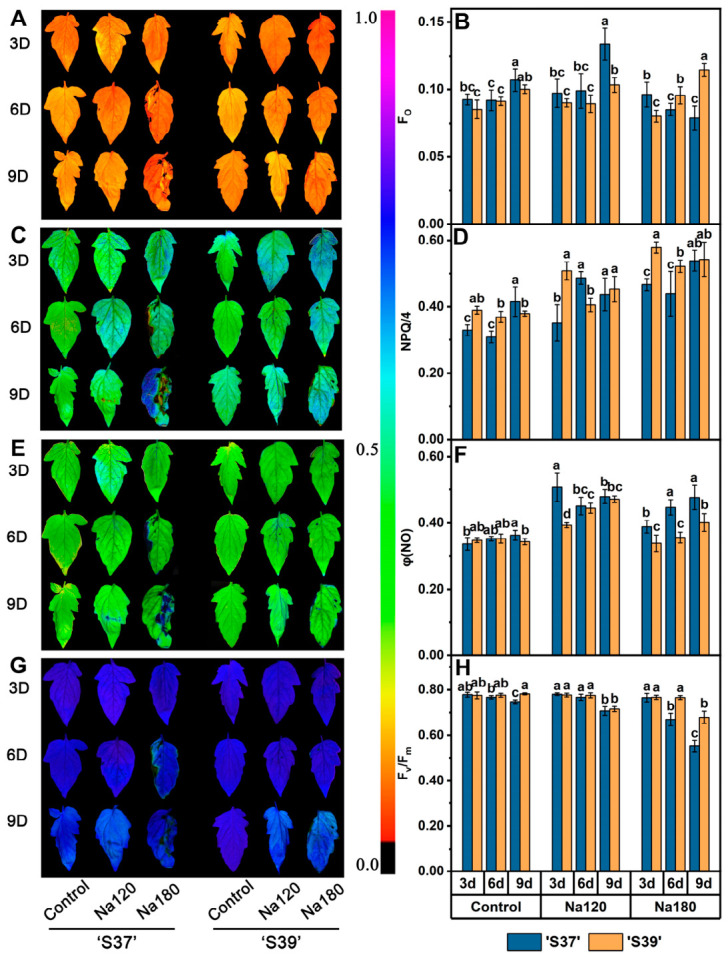
Chlorophyll fluorescence results and imaging photographs of processing tomato leaves under different NaCl treatment conditions. Note: Control, Na120, and Na180 represent Hoagland nutrient solution treatments containing 0, 120, and 180 mM NaCl, respectively. The treatment durations were 3, 6, and 9 days. ‘S37’ and S39’ represent salt-sensitive and salt-tolerant processing tomato genotypes, respectively. Fo: Minimal fluorescence (**A**,**B**); NPQ: Non-photochemical quenching (**C**,**D**); φ(NO): Quantum yield of non-regulated energy dissipation (**E**,**F**); F_v_/F_m_: Maximum quantum yield of photosystem II (**G**,**H**). To facilitate imaging, NPQ values were scaled by NPQ/4 to confine the range between 0 and 1. Each treatment had five biological replicates. Statistical significance was determined by one-way ANOVA followed by Duncan’s multiple. A *p*-value < 0.05 was considered statistically significant, and significant differences are indicated by different letters.

Fluorescence imaging of the indicators showed that, on day 6 of Na180 treatment, visible damage appeared at the leaf edges of ‘S37’. By day 9, the leaves exhibited unevenly distributed colors, and the right half showed deformation, likely due to dehydration-induced shrinkage and curling under high-concentration salt stress. In contrast, ‘S39’ only showed slight visible damage at the leaf edges on day 9 of Na180 treatment (Figure 6A,C,E,G). Throughout the process, the changes in the values of each indicator were largely consistent with the fluorescence imaging results. Under Na120 treatment, the Fo of ‘S37’ increased significantly by 25% compared with the control on day 9 and was significantly higher than that of ‘S39’ (Figure 6B), indicating that, under long-term low-salt treatment, the PSII reaction centers of ‘S37’ were more severely damaged. Under Na180 treatment, the Fo of ‘S37’ gradually decreased, while ‘S39’ showed the opposite trend. On day 3 of both Na120 and Na180 treatments, the NPQ of ‘S37’ was significantly lower than that of ‘S39’ (Figure 6D), suggesting that ‘S39’ could more rapidly activate non-photochemical quenching to enhance thermal dissipation and mitigate photoinhibition damage under NaCl treatment. On day 9 of NaCl treatment, although there was no significant difference in NPQ values between ‘S37’ and ‘S39’, fluorescence imaging revealed that prolonged NaCl treatment had caused substantial photosynthetic damage in ‘S37’ leaves (Figure 6C), indicating that both genotypes might have reached the limit of photoprotection. Under Na120 treatment, φ(NO) increased significantly in ‘S37’ on day 3 (Figure 6F), indicating that ‘S37’ was more sensitive to photoinhibition. Under Na180 treatment, φ(NO) increased significantly in both genotypes, but ‘S39’ exhibited significantly lower φ(NO) than ‘S37’, demonstrating its stronger ability to cope with salt stress. On days 6 and 9 of Na180 treatment, the F_v_/F_m_ of ‘S39’ was significantly higher than that of ‘S37’. In summary, under low-concentration NaCl treatment, ‘S39’ initiated thermal dissipation protection through moderately elevated φ(NO), while under high-concentration NaCl treatment, it rapidly activated the thermal dissipation mechanism via non-photochemical quenching (NPQ), converting excess light energy into heat to effectively reduce photodamage and better maintain F_v_/F_m_. In contrast, ‘S37’ showed insufficient NPQ in the early stages of salt stress, leading to a gradual increase in φ(NO) and a greater decline in F_v_/F_m_ (Figure 6F), reflecting higher sensitivity to salt stress and more severe leaf damage.

### 3.8. Effects of NaCl on Fast Chlorophyll Fluorescence Induction Kinetics Curves

#### 3.8.1. OJIP Curves

**Figure 7:**
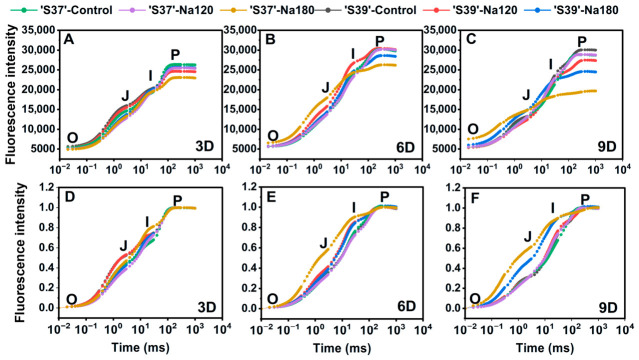
The fluorescence induction (PF) curves (**A**–**C**) and their O-P phase simple-normalized curves (**D**–**F**) using the parameter W_O-P_ = [(Ft − F_O_)/(Fm − F_O_)] were determined in processing tomato leaves under different NaCl treatment conditions. Control, Na120, and Na180 represent Hoagland nutrient solution treatments containing 0, 120, and 180 mM NaCl, respectively. The treatment durations were 3, 6, and 9 days. ‘S37’ and ‘S39’ represent salt-sensitive and salt-tolerant processing tomato genotypes, respectively. Each treatment had three biological replicates.

Compared with the control, Na120 treatment had a relatively minor impact on both genotypes (Figure 7A–C). Only the J-phase of ‘S37’ on day 3 and the P-phase of ‘S39’ on day 9 showed significant reductions relative to the control (Figure 7A). On day 3 of Na180 treatment, the O-phase and J-phase of ‘S37’ and ‘S39’ decreased significantly by 8.69%, 10.86% and 10.29%, 16.25%, respectively, compared with the control. The P-phase of ‘S37’ decreased significantly, while that of ‘S39’ showed no significant difference from the control. This indicates that ‘S39’ could more actively activate the photosynthetic system, reducing the O-phase and J-phase to counteract NaCl stress, thereby mitigating damage to the maximum photochemical efficiency of PSII (P-phase). On day 6, the O and J phases of ‘S37’ increased significantly, while its P phase decreased significantly compared with the control (Figure 7B), whereas ‘S39’ showed no significant difference from the control. This suggests that the damage to ‘S37’ was intensifying, while ‘S39’ may have restored its parameters to levels comparable to the control due to its strong inherent recovery capacity. On day 9, the O and J phases of ‘S37’ increased significantly, while its I phase decreased significantly compared with the control (Figure 7C). The P-phases of ‘S37’ and ‘S39’ decreased significantly by 31.86% and 18.10%, respectively, relative to the control. This indicates that irreversible damage occurred at the PSII reaction center of ‘S37’ (O-phase), severe obstruction appeared in the electron transfer chain from Q_A_ to Q_B_ on the acceptor side (I-phase), the maximum photochemical efficiency of PSII was severely impaired (P-phase), and the photosynthetic system gradually collapsed.

Simple normalizing the OJIP curves helps clarify changes in the electron transport chain on the acceptor side of PSII (Figure 7D–F). On day 3 of Na180 treatment (Figure 7D), the J–I phase of ‘S37’ and the O–I phase on day 6 (Figure 7E) showed substantial increases compared with the control, while the O–J phase of ‘S39’ on day 3 of Na180 treatment was lower than that of the control. On day 9 of Na180 treatment, the O–I phase of both ‘S37’ and ‘S39’ increased significantly compared with the control (Figure 7F), with ‘S37’ showing a greater increase than ‘S39’. Under Na120 treatment, the W_O-P_ phase showed no changes compared with the control, indicating that both genotypes exhibited high adaptability to Na120 treatment.

#### 3.8.2. L-Band

**Figure 8:**
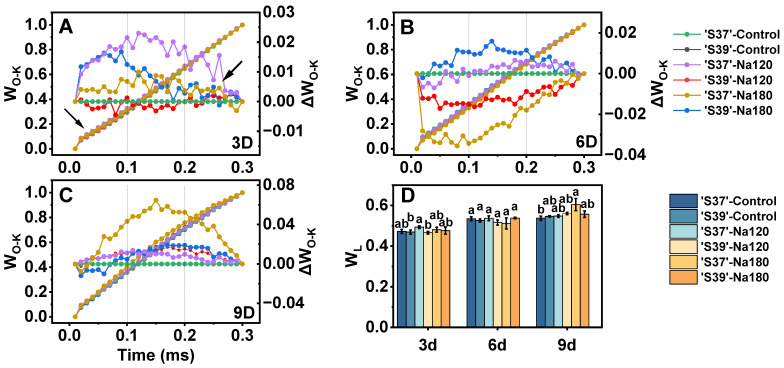
Normalized prompt fluorescence (PF) curves in the O–K phase. The area between the two dashed lines is the L-band. The left arrows indicate simple normalization, with the formula defined as: W_O-K_ = (F_t_ − F_O_)/(F_K_ − F_O_), the right arrows indicate double normalization, with the formula defined as: ΔW_O-K_ = W_O-K treatment_ − W_O-K control_ (**A**–**C**). The ratio of variable fluorescence F_K_ to the amplitude F_J_-F_O_, expressed as W_L_ = (F_L_ − F_O_)/(F_K_ − F_O_) (**D**). Control, Na120, and Na180 represent Hoagland nutrient solution treatments containing 0, 120, and 180 mM NaCl, respectively. The treatment durations were 3, 6, and 9 days. ‘S37’ and ‘S39’ represent salt-sensitive and salt-tolerant processing tomato genotypes, respectively. Each treatment had three biological replicates. Statistical significance was determined by one-way ANOVA followed by Duncan’s multiple. A *p*-value < 0.05 was considered statistically significant, and significant differences are indicated by different letters.

The region of the OJIP curve at 100–200 μs is referred to as the L-band. An increase in the L-band indicates reduced energy connectivity between PSII units [56]. To observe the L-band, the O–K phase of the OJIP curve was simple normalized (as shown by the left arrow curves in Figure 8A–C), but this failed to clearly reflect differences among treatments. Therefore, double normalization of the O–K phase was performed, making the L-band visible through difference transient curves (as shown by the right arrow curves in Figure 8A-C). The proportion of variable fluorescence at the L point (F_L_) to the amplitude F_k_–F_O_ (W_L_) was calculated (Figure 8D). Only ‘S37’ under Na180 treatment on day 9 showed a significant increase in W_L_ compared with the control, while no significant differences were observed between the two genotypes and the control under other treatments.

#### 3.8.3. K-Band

**Figure 9:**
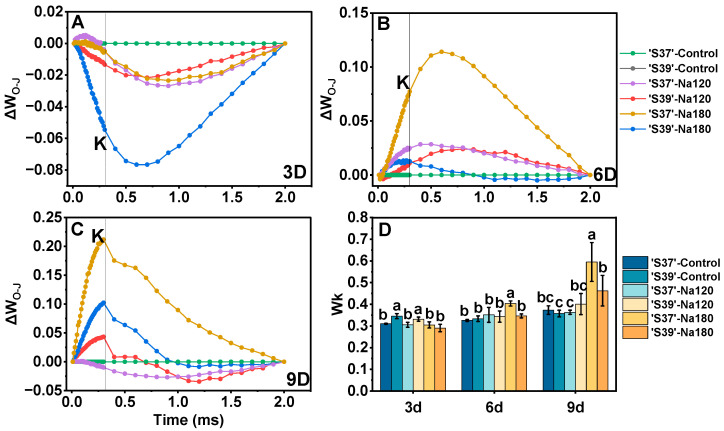
The differential analysis of OJIP kinetics (ΔW_O-J_) of the chlorophyll a fluorescence transient was obtained by normalizing the relative values between the O and J steps (W_O-J_) in the chlorophyll a fluorescence induction curves measured on processing tomato leaves under different salt concentrations. The intersection of each curve with the dashed line represents the K point. The formulas involved in double normalization are W_O-J_ = (F_t_ − F_O_)/(F_J_ − F_O_) and ΔW_O-J_ = W_O-J treatment_ − W_O-J control_ (**A**–**C**). The ratio of variable fluorescence F_K_ to the amplitude F_J_-F_O_ expressed as Wk = (F_k_ − F_O_)/(F_J_ − F_O_) (**D**). Control, Na120, and Na180 represent Hoagland nutrient solution treatments containing 0, 120, and 180 mM NaCl, respectively. The treatment durations were 3, 6, and 9 days. ‘S37’ and ‘S39’ represent salt-sensitive and salt-tolerant processing tomato genotypes, respectively. Each treatment had three biological replicates. Statistical significance was determined by one-way ANOVA followed by Duncan’s multiple. A *p*-value < 0.05 was considered statistically significant, and significant differences are indicated by different letters.

The region of the OJIP curve at 150–200 μs is referred to as the K-band [56]. An increase in the K-band indicates inhibition or inactivation of the oxygen-evolving complex (OEC). To observe the K-band, the O–J phase of the OJIP curve was double normalized (Figure 9A–C), and the proportion of variable fluorescence at the K point (F_k_) to the amplitude F_J_–F_o_ (W_k_) was calculated (Figure 9D). On day 3, the K-band and W_k_ of ‘S39’ under Na180 treatment were significantly lower than those of the control. On day 6, only ‘S37’ under Na180 treatment showed a significantly higher W_k_ than the control. On day 9 of Na180 treatment, both genotypes exhibited a K inflection point, with the W_k_ of ‘S37’ being significantly higher than that of ‘S39’.

#### 3.8.4. G-Band

**Figure 10:**
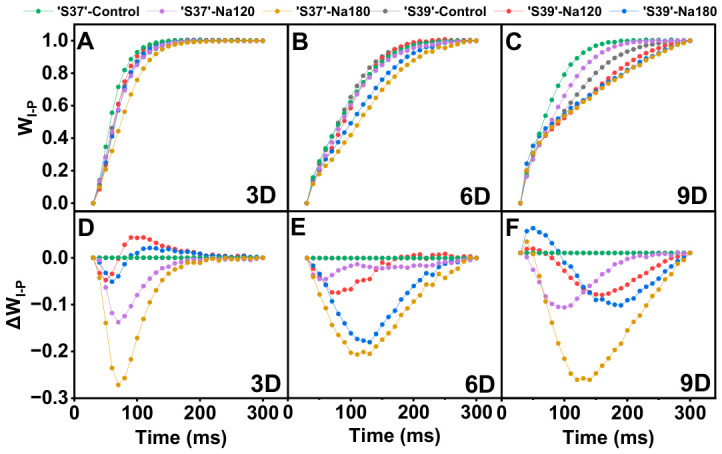
The differential analysis of OJIP kinetics (ΔW_I-P_) of the chlorophyll a fluorescence transient was obtained by normalizing the relative values between the I and P steps (W_I-P_) in the chlorophyll a fluorescence induction curves measured on processing tomato leaves under different salt concentrations. The formulas involved in simple normalization is W_I-P_ = (F_t_ − F_I_)/(F_P_ − F_I_) (**A**–**C**) and that for double normalization is ΔW_I-P_ = W_I-P treatment_ − W_I-P control_ (**D**–**F**). Control, Na120, and Na180 represent Hoagland nutrient solution treatments containing 0, 120, and 180 mM NaCl, respectively. The treatment durations were 3, 6, and 9 days. ‘S37’ and ‘S39’ represent salt-sensitive and salt-tolerant processing tomato genotypes, respectively. Each treatment had three biological replicates. Statistical significance was determined by one-way ANOVA followed by Duncan’s multiple.

The G-band reflects the status of electron transfer from the plastoquinone (PQ) pool to the acceptor side of PSI [57]. To observe the G-band, the I-P segment of the OJIP curve was double normalized (Figure 10A–C). As the treatment duration increased, the differences between Na120 and Na180 treatments compared with the control gradually widened. Under the same treatment duration, the difference between Na120 and the control was relatively minor. To better analyze this variation, differential kinetic analysis was performed on the I-P phase of the OJIP curve (Figure 10D–F). Under Na120 treatment, the fluctuation amplitude for both genotypes was smaller than that under Na180 treatment. Furthermore, the fluctuation amplitude of ‘S37’ was consistently greater than that of ‘S39’, indicating that the reaction centers of the photosystem of ‘S39’ possess higher stability.

#### 3.8.5. Effects of NaCl on OJIP-Test Parameters

**Figure 11:**
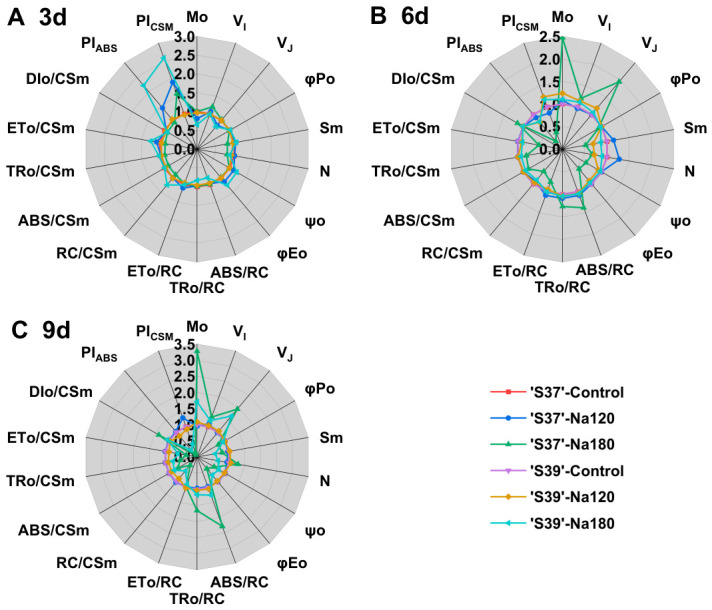
Analysis of JIP parameters derived from the OJIP test for processing tomato leaves under different NaCl treatments on days 3 (**A**), 6 (**B**), and 9 (**C**). For each measured parameter, values were simple normalized relative to the control (0 mM NaCl) at the same timepoint. Control, Na120, and Na180 represent Hoagland nutrient solution treatments containing 0, 120, and 180 mM NaCl, respectively. The treatment durations were 3, 6, and 9 days. ‘S37’ and ‘S39’ represent salt-sensitive and salt-tolerant processing tomato genotypes, respectively. Each treatment had three biological replicates. Statistical significance was determined by one-way ANOVA followed by Duncan’s multiple. A p-value < 0.05 was considered statistically significant, and significant differences are indicated by different letters. Significant differences in radar plots are shown in Supplementary Table S2. Note: Mo, initial slope of the OJIP fluorescence induction curve (off rate of RCs); V_I_, variable fluorescence at point I; V_J_, relative variable fluorescence at point J; φPo, the maximum quantum yield of primary PSII photochemistry; S_m_, area between the normalized OJIP; N, the number of Q_A_ redox turnovers before reaching Fm; Ψ_O_, the probability with which a PSII-trapped electron is transferred from Q_A_ to Q_B_ in the electron transport chain; φE_O_, the quantum yield of electron transport flux from Q_A_ to Q_B_; ABS/RC, light energy absorbed per reaction center; TR_O_/RC, energy captured per reaction center for reduction of Q_A_ (at t = F_O_); ET_O_/RC, energy captured per reaction center for electron transfer (at t = F_O_); RC/CS_m_, number of active reaction centers per unit area (at t = F_m_); ABS/CS_m_, light energy absorbed per unit area (at t = F_m_); TR_O_/CS_m_, light energy captured per unit area (at t = F_m_); ET_O_/CS_m_, quantum yield per unit area for electron transfer (at t = F_m_); DI_O_/CS_m_, heat dissipation per unit area (at t = F_m_); PI_ABS_, performance index on absorption basis; PI_csm_, performance Index on cross section basis.

To characterize the differences in the dynamic tolerance (at 3, 6, and 9 days) of PSII in seedlings of two processing tomato germplasms with different salt tolerances to continuous NaCl treatment, the OJIP curve was mathematically analyzed to derive JIP-test-related parameters. After the simple normalizing of each parameter against the control treatment, radar charts were plotted (Figure 11), and the analysis of significant differences is shown in Supplementary Table S2. Under Na120 treatment, the parameters of ‘S39’ remained relatively stable, all showing no significant difference compared with the control. For ‘S37’, only on day 6 did the various parameters show no significant difference from the control. On day 3 of Na180 treatment, V_I_ of ‘S37’ increased significantly compared with the control (Figure 11A), while S_m_, N, ABS/CS_m_, TR_O_/CS_m_, and DIo/CSm decreased significantly. For ‘S39’, φP_O_, ψ_O_, φE_O_, ABS/RC, RC/CS_m_, ET_O_/CS_m_, PI_ABS_, and PI_CSM_ significantly increased compared with the control, while M_O_, V_J_, N, TR_O_/RC, and DIo/CSm significantly decreased. On day 6 of the Na180 treatment (Figure 11B), only some parameters of ‘S37’ showed significant differences compared with the control, while all parameters of ‘S39’ exhibited no significant differences from the control. On day 9 of Na180 treatment (Figure 11C), M_O_, V_I_, V_J_, N, ABS/RC, TR_O_/RC, and DIo/CS_m_ of ‘S37’ were significantly higher than both the control and ‘S39’, while φP_O_, S_m_, ψ_O_, φE_O_, RC/CS_m_, ABS/CS_m_, TR_O_/CS_m_, ET_O_/CS_m_, and PI_ABS_ were significantly lower than the control and ‘S39’. In summary, under NaCl treatment, the JIP-test parameters of ‘S39’ were more stable than those of ‘S37’. Please refer to Supplementary Table S1 for the equations and definitions of the JIP-test parameters.

### 3.9. Correlation Analysis

**Figure 12:**
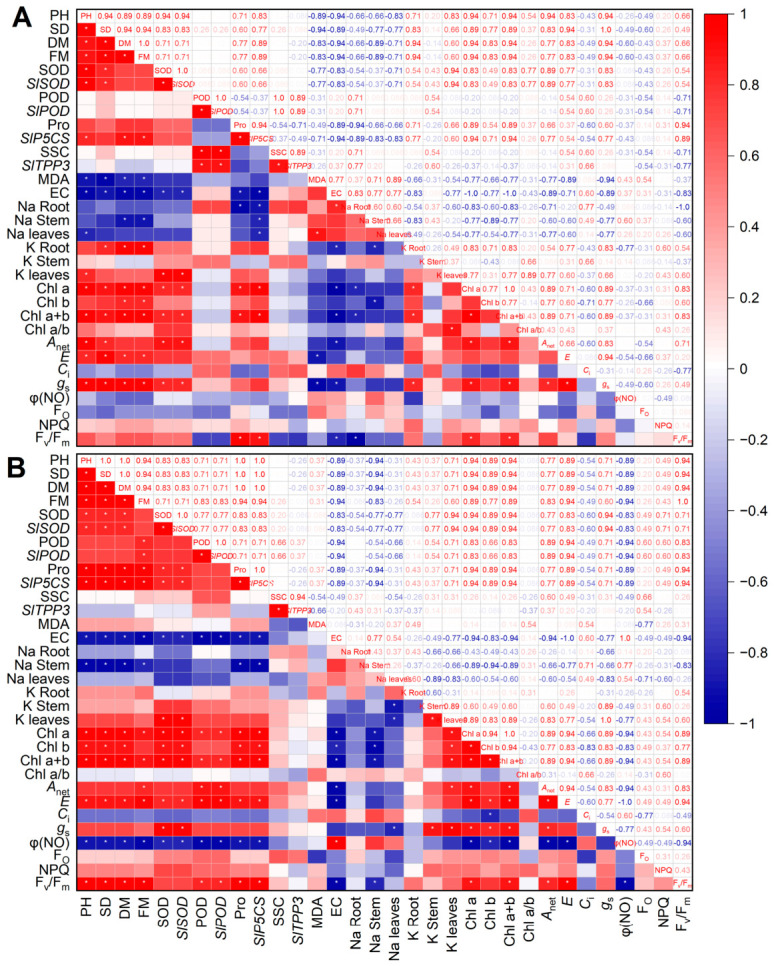
After simple normalization with reference to the control, Spearman correlation analyses were performed for Na120 (**A**) and Na180 (**B**). Note: PH, plant height; SD, stem diameter; DM, dry mass; FM, fresh mass; SOD, SOD activity; *SlSOD1*, *SlSOD1* expression level; POD, POD activity; *SlPOD1*, *SlPOD1* expression level; Pro, proline content; *SlP5CS*, *SlP5CS* expression level; SSC, soluble sugar content; *SlTPP3*, *SlTPP3* expression level; MDA, malondialdehyde content; EC, electrical conductivity; Na Root, Na content in roots; Na Stem, Na content in stems; Na leaves, Na content in leaves; K Root, K content in roots; K Stem, K content in stems; K leaves, K content in leaves; Chl a, chlorophyll a content; Chl b, chlorophyll b content; Chl a + b, chlorophyll a + b content; Chl a/b, chlorophyll a/b content; *A*_net_, net photosynthetic rate; *E*, transpiration rate; *C*_i_, intercellular CO_2_ concentration; *g*_s_, stomatal conductance; φ(NO), quantum yield of non-regulated energy dissipation; F_o_, minimum fluorescence emitted when all PSII reaction centers are open and recorded; NPQ, non-photochemical quenching; F_v_/F_m_, maximum quantum yield of photosystem II. * indicates a significant correlation (*p* < 0.05).

To investigate the correlations among major indicators under NaCl treatment, the data were normalized to the control values, and Spearman correlation analyses were performed separately for Na120 (Figure 12A) and Na180 (Figure 12B). The results showed that under both Na120 and Na180 treatments, plant height, stem diameter, dry mass, and fresh mass were significantly positively correlated with chl a content, chl a + b content, and transpiration rate. Under Na120 treatment, these growth indicators were significantly negatively correlated with malondialdehyde content and electrical conductivity. Under Na180 treatment, growth indicators were additionally significantly negatively correlated with stem Na content and φ(NO). These findings indicate that the inhibitory effect of NaCl on processing tomato growth is associated with membrane lipid peroxidation, and that, under Na180 treatment, Na accumulation in stems and increased non-regulated energy dissipation of photosystem II further aggravated growth inhibition. SOD and POD activities were significantly positively correlated with *SlSOD1* and *SlPOD1* expression levels, respectively; proline and soluble sugar contents were significantly positively correlated with *SlP5CS* and *SlTPP3* expression levels, respectively. This suggests that NaCl treatment may up-regulate the expression of the corresponding genes to enhance antioxidant enzyme activities and promote the synthesis of osmotic regulatory substances, thereby scavenging reactive oxygen species and maintaining osmotic balance. Under both NaCl concentrations, electrical conductivity was significantly negatively correlated with growth indicators, SOD content, *SlSOD1* expression, proline content, and *SlP5CS* expression. Under Na180 treatment, electrical conductivity was also significantly negatively correlated with POD activity and *SlPOD1* expression. These results further indicate that activation of the antioxidant and osmoregulatory systems helps alleviate oxidative damage and mitigate the inhibitory effect of NaCl on plant growth. Furthermore, under NaCl treatment, proline content and *SlP5CS* expression were significantly positively correlated with chl a content and chl a + b content. Under Na180 treatment, these were also significantly positively correlated with chl b content. This suggests that osmotic regulatory substances may protect the stability of the photosynthetic system by maintaining leaf water status.

## 4. Discussion

### 4.1. Effects of NaCl on Growth and Physiological Characteristics of Processing Tomato with Different Salt Tolerances

NaCl is the most common component in salt stress. The most direct effect of NaCl on plants often manifests as a significant inhibition of the increase in plant height, stem diameter, and dry and fresh mass, among others [58,59]. However, genotypes with different salt tolerances exhibit significant differences in their tolerance to salt stress. Previous studies have shown that salt stress inhibits the growth of salt-tolerant genotypes to a significantly lesser extent than that of salt-sensitive genotypes [60,61]. Similar results were observed in the present study. Compared with the control, NaCl significantly reduced the dry mass of ‘S37’, whereas the dry mass of ‘S39’ showed no significant difference from the control at 6 and 9 days of NaCl treatment. This study uses whole-plant fresh mass as an indicator of plant water status under salt stress, which effectively reflects differences in overall water status and growth tolerance among genotypes [62]. Relative leaf water content, used in related studies [63,64], can serve as a reference indicator for subsequent experiments. Combining whole-plant fresh mass with relative leaf water content allows for a more comprehensive evaluation of leaf water status under salt stress.

The inhibition of plant growth occurs through two consecutive phases. First, NaCl reduces the water absorption capacity of plant roots, causing osmotic stress and leading to physiological drought in plants. Plants synthesize and accumulate proline and soluble sugars to alleviate the osmotic imbalance induced by NaCl stress [65,66,67,68]. Second, excess Na enters the plant and affects growth through ionic imbalance and inhibition of photosynthesis. Relative electrical conductivity and malondialdehyde (MDA) are considered important indicators for evaluating the degree of damage to plant cell membranes under stress conditions [69,70]. The purpose of increasing plant salt tolerance can be achieved by enhancing antioxidant enzyme activity [71]. In the present study, under Na120 and Na180 treatments, MDA content in ‘S39’ initially increased and then decreased, suggesting that, after transient oxidative damage, the defense system of ‘S39’ was activated, alleviating membrane lipid peroxidation. In contrast, MDA content and relative electrical conductivity in ‘S37’ remained consistently high under Na180 treatment with no significant changes. In combination with the significant decrease in SOD and POD activities, this indicates that the capacity of ‘S37’ to scavenge reactive oxygen species (ROS) was diminished, resulting in severe cell membrane damage. Under Na180 treatment, both SOD and POD activities were significantly higher in ‘S39’ than in ‘S37’. These results are consistent with those reported by Ji et al. [72] in walnut, the increases in SOD and POD activities relative to the control were greater in the salt-tolerant genotype than in the salt-sensitive genotype, while the MDA content and electrical conductivity values showed the opposite trend. Furthermore, under Na180 treatment, the number of significant correlation pairs was higher, indicating that processing tomato activates a more complex salt tolerance mechanism under Na180 treatment, and that Na180 treatment is more effective in revealing differences among different genotypes.

### 4.2. Effects of NaCl on Photosynthetic Characteristics of Processing Tomato with Different Salt Tolerances

Photosynthesis, as the physiological basis for plant growth, is an important indicator for evaluating plant growth adaptability [73]. In this study, under salt stress, the reduction in photosynthetic pigment content was smaller in ‘S39’ than in ‘S37’, indicating that photosynthetic pigments in ‘S39’ were more stable under salt stress. Begum et al. [74] reported similar results and concluded that salt stress accelerates the degradation of photosynthetic pigments. In the present study, at 9 days under Na180 treatment, *C*_i_ in ‘S37’ increased significantly by 90.17% compared with the control, while *g*_s_ decreased significantly by 81.16%, indicating that the primary factor limiting photosynthesis in ‘S37’ had shifted to non-stomatal limitation. In contrast, *C*_i_ in ‘S39’ showed no significant difference from the control, whereas *g*_s_ decreased significantly by 63.93%, suggesting that ‘S39’ responded to salt stress by closing stomata to reduce water loss. These results are consistent with previous studies under drought, heat, and salt stress, in which plants actively close their stomata to reduce water loss and maintain the integrity of the photosynthetic system [75,76], representing a typical stress-tolerance strategy [77].

The OJIP curve can accurately reflect changes in the primary photochemical reaction of PSII reaction centers, electron transport in the photosynthetic apparatus, and related processes [78]. Previous studies have demonstrated that salt stress damages PSII and causes significant changes in chlorophyll fluorescence parameters [79,80], which is consistent with the results of the present study. In this study, under Na120 treatment, W_k_ showed no significant changes in either genotype, indicating that this concentration did not cause severe damage to PSII. Under Na180 treatment, the O and J steps in ‘S37’ increased significantly, while the I and P steps decreased markedly at 6 and 9 days compared with the control. Moreover, the K-band, L-band, V_I_, and V_J_ increased, whereas ψo and φEo decreased, indicating severe damage to the PSII reaction centers, electron transport from Q_A_ to Q_B_, and the oxygen-evolving complex (OEC), as well as thylakoid dissociation and aggravated photoinhibition [81]. At most timepoints, the variation ranges of OJIP-related parameters were smaller in ‘S39’ than in ‘S37’, indicating greater stability of the photosynthetic electron transport chain and photosystem in ‘S39’. Furthermore, under Na180 treatment at 3 days, W_k_ in ‘S39’ decreased significantly compared with the control, suggesting that ‘S39’ can alleviate early salt stress by regulating OEC activity or enhancing electron flow. This is consistent with previous findings, where the chlorophyll fluorescence parameters of salt-tolerant mulberry grafted seedlings were less damaged by salt stress than those of salt-sensitive own-rooted seedlings [82].

### 4.3. Effects of NaCl on Elemental Contents in Roots, Stems, and Leaves of Processing Tomato with Different Salt Tolerances

The absorption of mineral elements by plants aids in coping with environmental stress. Under NaCl treatment, excessive accumulation of Na and Cl can lead to inhibited K absorption [83], greatly affecting the absorption and transport of mineral elements. Many studies have shown that NaCl treatment significantly increases Na content in plant tissues and reduces the K/Na, Mg/Na, and Ca/Na ratios in roots, stems, and leaves [84,85], severely disrupting ion homeostasis and leading to metabolic disorders and even death [86]. In this study, under Na120 treatment on day 9 and under Na180 treatment, the Na accumulation in the stems of ‘S39’ was significantly higher than that in ‘S37’, indicating that ‘S39’ could sequester Na in the stems, adopting an ‘exclusion’ strategy to reduce Na transport to leaves and maintain normal physiological activities such as photosynthesis. This is consistent with previous findings [87,88]. However, studies by Zhang et al. [89] in mulberry and Wu et al. [84] in wheat demonstrated that retaining Na in the roots is also an effective strategy for mitigating Na toxicity in plants, suggesting that salt tolerance strategies vary among plant species and may be closely associated with species and genotype. Previous studies have shown that maintaining mineral element transport rates is important for maintaining ion homeostasis in plants, providing scientific support for its role as a key factor in salt tolerance [90]. In the present study, on day 3 of NaCl treatment, S_K,Na_, S_Ca,Na_, and S_Fe,Na_ from roots to leaves in ‘S39’, and S_K,Na_ from roots to stems, were all higher than those in ‘S37’, indicating that ‘S39’ could rapidly promote the transport of key elements such as K (photosynthesis), Ca (signaling molecules), and Fe (chlorophyll synthesis) to the above-ground parts in tolerance to early NaCl treatment. The transport capacity of ‘S37’ weakened on day 9, with the greatest decline observed under Na180 treatment. At this time, ‘S39’ could still maintain a relatively high transport rate of mineral elements to maintain element homeostasis, which might be the key to its salt tolerance. However, the NaCl treatment duration in this study was relatively short, and longer-term NaCl treatments will be carried out in the future to validate salt tolerance over the whole growth period. Furthermore, the NaCl concentrations of 120 and 180 mM used in this study are both relatively high stress levels. Although this helped amplify the differences between the genotypes, it limited the analysis of their differential tolerance under moderate NaCl stress. In future studies, we will consider including a wider range of NaCl concentrations (e.g., 30–100 mM) to more comprehensively elucidate the salt tolerance mechanisms. Meanwhile, in future measurements of photosynthetic parameters, we will use the actual atmospheric CO_2_ concentration as the set value to better reflect photosynthetic performance under real environmental conditions.

## 5. Conclusions

**Figure 13:**
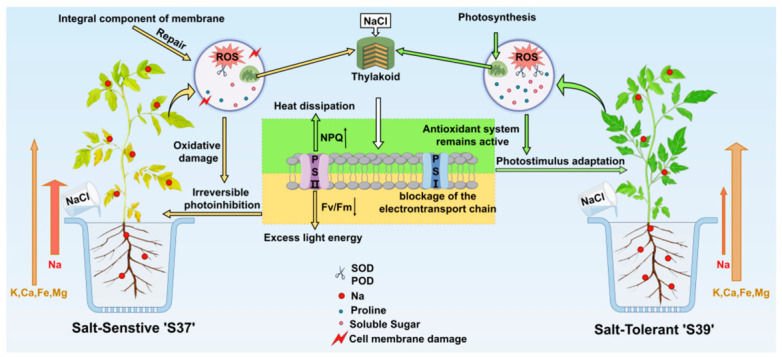
Schematic diagram of the tolerance differences of the two genotypes to NaCl [(this picture is drawn by Figdraw (https://www.figdraw.com/#/paint_canvas_v2?canva=2155637, accessed on 5 May 2026)]. Under NaCl stress, ‘S39’ employs multiple strategies to cope, including enhancing antioxidant enzyme activity, adopting a “salt exclusion” strategy to sequester Na in the roots and stems, maintaining efficient transport of K, Mg, Ca, and Fe to the above-ground parts, and activating the NPQ photoprotection mechanism. In contrast, under salt stress, ‘S37’ exhibits reactive oxygen species (ROS) accumulation, leading to cell membrane damage, blockage of photosynthetic electron transport, and irreversible photosynthetic damage. Moreover, its weaker ion transport capacity results in more severe salt injury.

NaCl inhibited the growth of both genotypes, with fresh mass being the most sensitive to salt stress. This further validates the idea that fresh mass is a key phenotypic indicator reflecting the degree of growth inhibition. Under the same NaCl stress conditions, the salt-sensitive genotype ‘S37’ exhibited wilting earlier than the salt-tolerant genotype ‘S39’. During the early stage of NaCl stress (3 d), ‘S39’ exhibited higher levels of tolerance than ‘S37’ in terms of the antioxidant enzyme system (SOD activity), osmolyte accumulation (proline content), and photoprotection system (NPQ), whereas the corresponding tolerance of ‘S37’ was relatively weaker than that of ‘S39’. Under long-term high-concentration NaCl (Na180, 9 d) treatment, ‘S39’ exhibited a salt exclusion strategy, sequestering Na in roots and stems. ‘S39’ exhibited higher translocation efficiencies of K, Mg, Ca and Fe than ‘S37’ under NaCl stress at most timepoints and salinity levels, which contributed to the reconstruction of mineral nutrient balance in above-ground tissues. ‘S39’ shows more stable photosynthetic system indicators than ‘S37’, including chlorophyll content, photosynthetic parameters, and chlorophyll fluorescence parameters (Figure 13**)**. In summary, ‘S39’ formed a multi-layered salt tolerance strategy through efficient coordination of antioxidant enzyme systems, osmotic regulators, and mineral element balance, ultimately maintaining better growth status and physiological functions under salt stress.

## Figures and Tables

**Table 1:** Primers for qRT-PCR.

Gene Name	Forward Primer (5′-3)	Reverse Primer (5′-3)
*Actin*	ATCTTGGCTTCCCTCAGCAC	GCATCTCTGGTCCAGTAGGAAAT
*SlSOD1*	AACCTGGACTTCATGGCTTC	CCAGCAGGATTGTAATGTGG
*SlPOD1*	GAGAGGTCTGTTCCAATCCGATGC	TTCGTTGAGTGGTCCATCTACAAGC
*SlTPP3*	TTCACTCATTCCATGGCTCGT	CTGGGAAAGTTTGGCTTGGTG
*SlP5CS*	TGCTGTAGGTGTTGGTCGTCA	TGCCATCAAGCTCAGTTTGTG

**Table 2:** Effects of different NaCl treatments on growth indicators of processing tomatoes.

Variety	Treatment	Plant Height (cm)			Stem Diameter (mm)			Dry Mass (g)			Fresh Mass (g)		
		3 d	6 d	9 d	3 d	6 d	9 d	3 d	6 d	9 d	3 d	6 d	9 d
‘S37’	Control	18.73 ± 1.10 a	25.13 ± 0.96 a	30.17 ± 1.80 a	5.40 ± 0.26 ab	5.73 ± 0.21 a	6.63 ± 0.23 a	8.23 ± 0.15 a	8.70 ± 0.46 a	9.90 ± 0.56 a	15.37 ± 2.82 a	24.63 ± 5.20 a	56.07 ± 5.46 a
	Na120	16.07 ± 1.10 cd	19.77 ± 1.57 bc	23.43 ± 2.02 b	4.77 ± 0.25 c	4.90 ± 0.10 c	5.53 ± 0.06 b	7.90 ± 0.17 b	8.10 ± 0.10 b	8.33 ± 0.23 b	8.93 ± 1.79 c	12.73 ± 1.46 b	26.90 ± 3.70 b
	Na180	14.30 ± 0.56 e	16.77 ± 1.23 c	18.53 ± 0.68 c	4.67 ± 0.12 c	4.77 ± 0.12 c	4.83 ± 0.06 c	7.57 ± 0.15 c	7.77 ± 0.15 b	7.87 ± 0.06 b	8.60 ± 1.60 c	10.20 ± 0.60 b	13.67 ± 0.91 c
‘S39’	Control	18.03 ± 1.05 ab	26.13 ± 2.51 a	31.13 ± 1.58 a	5.63 ± 0.15 a	5.90 ± 0.40 a	6.50 ± 0.26 a	8.03 ± 0.25 ab	8.20 ± 0.36 b	8.93 ± 1.22 b	13.73 ± 2.75 ab	23.23 ± 1.72 a	52.40 ± 5.90 a
	Na120	17.13 ± 0.35 bc	20.93 ± 2.18 b	23.97 ± 0.50 b	5.23 ± 0.23 ab	5.33 ± 0.21 b	5.67 ± 0.03 b	7.93 ± 0.15 ab	8.00 ± 0.26 b	8.30 ± 0.10 b	10.90 ± 1.97 bc	14.00 ± 0.53 b	25.70 ± 2.95 b
	Na180	14.73 ± 0.47 de	18.37 ± 0.93 bc	20.87 ± 0.60 c	5.00 ± 0.26 bc	5.07 ± 0.15 bc	5.10 ± 0.10 c	7.53 ± 0.21 c	7.57 ± 0.06 b	7.97 ± 0.17 b	8.53 ± 1.57 c	12.30 ± 0.26 b	15.97 ± 1.95 c

Note: Control, Na120, and Na180 represent Hoagland nutrient solution treatments containing 0, 120, and 180 mM NaCl, respectively, with treatment durations of 3, 6, and 9 days. ‘S37’ and ‘S39’ represent salt-sensitive and salt-tolerant processing tomato genotypes, respectively. The data in the table represent the mean ± standard deviation. Each treatment had three biological replicates. Statistical significance was determined by one-way ANOVA followed by Duncan’s multiple. A *p*-value < 0.05 was considered statistically significant, and significant differences are indicated by different letters.

**Table 3:** Effect of salt treatment on processing tomato seedlings S_k,Na_, S_Mg,Na_, S_Ca,Na_, S_Fe,Na_.

Time	Variety	Treatment	S_k,Na_		S_Mg,Na_		S_Ca,Na_		S_Fe,Na_	
			Root–Stem	Root–Leaf	Root–Stem	Root–Leaf	Root–Stem	Root–Leaf	Root–Stem	Root–Leaf
3 d	‘S37’	Control	1.23 ± 0.10 h	0.75 ± 0.08 efg	0.61 ± 0.08 ij	1.36 ± 0.13 ef	0.52 ± 0.05 efg	2.24 ± 0.30 d	0.22 ± 0.01 efg	2.58 ± 0.20 b
		Na120	1.73 ± 0.04 fg	0.91 ± 0.03 cdef	0.79 ± 0.01 hij	1.24 ± 0.05 fg	1.04 ± 0.02 cd	2.88 ± 0.08 bc	0.48 ± 0.02 ab	1.90 ± 0.01 c
		Na180	1.37 ± 0.29 gh	0.78 ± 0.17 cdefg	0.58 ± 0.02 j	1.04 ± 0.05 gh	0.45 ± 0.09 efg	1.65 ± 0.28 e	0.11 ± 0.01 ij	0.99 ± 0.09 d
	‘S39’	Control	1.22 ± 0.13 h	0.56 ± 0.06 g	0.66 ± 0.10 hij	1.07 ± 0.17 gh	0.50 ± 0.04 efg	1.47 ± 0.09 ef	0.42 ± 0.06 bc	1.73 ± 0.13 c
		Na120	2.09 ± 0.31 ef	0.77 ± 0.02 defg	0.88 ± 0.09 ghi	1.20 ± 0.03 fg	1.32 ± 0.13 c	3.11 ± 0.16 b	0.59 ± 0.01 a	5.73 ± 0.06 a
		Na180	1.47 ± 0.07 gh	0.73 ± 0.05 fg	0.58 ± 0.01 j	1.01 ± 0.04 gh	0.34 ± 0.02 efg	1.25 ± 0.09 efg	0.18 ± 0.01 fg	1.60 ± 0.08 c
6 d	‘S37’	Control	2.29 ± 0.29 cde	0.99 ± 0.14 cd	1.20 ± 0.11 ef	1.93 ± 0.1 7 bc	0.74 ± 0.07 de	2.37 ± 0.25 d	0.08 ± 0.05 ij	0.97 ± 0.28 d
		Na120	2.25 ± 0.37 cde	0.72 ± 0.09 fg	2.73 ± 0.40 a	1.69 ± 0.18 cd	2.18 ± 0.36 b	2.98 ± 0.66 b	0.40 ± 0.04 bcd	0.83 ± 0.11 def
		Na180	1.54 ± 0.09 gh	0.57 ± 0.02 g	1.31 ± 0.19 de	1.18 ± 0.10 fg	1.01 ± 0.18 cd	1.43 ± 0.65 ef	0.17 ± 0.03 fg	0.55 ± 0.27 efg
	‘S39’	Control	1.54 ± 0.22 gh	0.97 ± 0.01 cde	0.86 ± 0.08 ghi	1.57 ± 0.07 de	0.65 ± 0.11 def	2.47 ± 0.29 cd	0.01 ± 0.01 j	0.88 ± 0.11 de
		Na120	2.65 ± 0.40 bc	0.78 ± 0.04 cdefg	2.36 ± 0.22 b	1.58 ± 0.04 de	2.63 ± 0.85 a	4.02 ± 0.13 a	0.47 ± 0.20 ab	1.73 ± 0.11 c
		Na180	2.19 ± 0.27 de	1.27 ± 0.06 b	1.60 ± 0.13 c	2.42 ± 0.37 a	0.71 ± 0.24 de	2.38 ± 0.12 d	0.29 ± 0.12 cdef	1.66 ± 0.05 c
9 d	‘S37’	Control	1.65 ± 0.13 fgh	0.61 ± 0.00 g	1.05 ± 0.06 fg	1.21 ± 0.06 fg	0.31 ± 0.02 efg	0.58 ± 0.04 hi	0.33 ± 0.06 cde	0.70 ± 0.19 def
		Na120	2.54 ± 0.20 cd	0.63 ± 0.05 g	1.45 ± 0.12 cd	1.26 ± 0.04 fg	0.63 ± 0.05 defg	0.99 ± 0.12 fgh	0.48 ± 0.17 ab	0.99 ± 0.33 d
		Na180	3.04 ± 0.16 b	1.00 ± 0.05 c	0.76 ± 0.04 hij	0.83 ± 0.05 h	0.18 ± 0.01 g	0.38 ± 0.01 i	0.08 ± 0.00 ij	0.28 ± 0.02 g
	‘S39’	Control	1.71 ± 0.18 fg	0.77 ± 0.11 defg	0.77 ± 0.06 hij	1.18 ± 0.07 fg	0.21 ± 0.02 fg	0.77 ± 0.07 hi	0.18 ± 0.04 fg	0.50 ± 0.10 fg
		Na120	2.62 ± 0.36 bcd	0.89 ± 0.13 cdef	1.11 ± 0.12 efg	1.43 ± 0.19 def	0.43 ± 0.03 efg	1.28 ± 0.06 efg	0.26 ± 0.08 def	1.00 ± 0.29 d
		Na180	6.77 ± 0.11 a	4.13 ± 0.38 a	0.91 ± 0.01 gh	2.00 ± 0.22 b	0.18 ± 0.00 g	0.83 ± 0.08 ghi	0.10 ± 0.03 ij	0.55 ± 0.22 efg

Note: Control, Na120, and Na180 represent Hoagland nutrient solution treatments containing 0, 120, and 180 mM NaCl, respectively. The treatment durations were 3, 6, and 9 days. ‘S37’ and ‘S39’ represent salt-sensitive and salt-tolerant processing tomato genotypes, respectively. The S_X,Na_ value was calculated, where a higher S_X,Na_ indicates a stronger capacity of the plant to retain Na while transporting element X. Each treatment had three biological replicates. Statistical significance was determined by one-way ANOVA followed by Duncan’s multiple. A *p*-value < 0.05 was considered statistically significant, and significant differences are indicated by different letters.

**Table 4:** Effects of salt stress on the photosynthetic gas exchange parameters of processed tomato seedlings.

Variety	Treatment	*A*_net_ (µmol m^−2^ s^−1^)			*E* (mmol m^−2^ s^−1^)			*C*_i_ (µmol mol^−1^)			*g*_s_ (mol m^−2^ s^−1^)		
		3 d	6 d	9 d	3 d	6 d	9 d	3 d	6 d	9 d	3 d	6 d	9 d
‘S37’	Control	9.87 ± 0.60 a	9.91 ± 0.67 a	8.99 ± 0.37 a	1.97 ± 0.14 a	2.18 ± 0.38 a	2.37 ± 0.09 a	257.35 ± 11.56 a	216.29 ± 12.26 b	206.71 ± 7.64 b	0.10 ± 0.01 a	0.11 ± 0.00 a	0.09 ± 0.00 a
	Na120	9.27 ± 0.66 a	7.67 ± 0.52 b	6.18 ± 0.68 b	1.35 ± 0.06 b	1.44 ± 0.31 b	1.41 ± 0.06 bc	158.05 ± 17.67 cd	163.36 ± 9.49 cd	189.06 ± 15.25 b	0.07 ± 0.01 b	0.06 ± 0.00 d	0.04 ± 0.01 b
	Na180	3.99 ± 1.70 c	2.48 ± 0.15 d	2.41 ± 0.27 c	0.81 ± 0.20 c	0.72 ± 0.14 c	0.46 ± 0.08 d	234.39 ± 8.60 b	372.34 ± 40.04 a	393.12 ± 17.08 a	0.05 ± 0.00 c	0.02 ± 0.01 e	0.02 ± 0.00 c
‘S39’	Control	10.03 ± 0.66 a	9.68 ± 1.23 a	9.13 ± 0.63 a	1.39 ± 0.11 b	1.42 ± 0.12 b	1.63 ± 0.19 b	154.87 ± 7.83 cd	187.76 ± 6.06 bc	158.20 ± 4.73 cd	0.11 ± 0.01 a	0.10 ± 0.01 b	0.08 ± 0.00 a
	Na120	9.37 ± 0.11 a	8.96 ± 0.82 a	8.04 ± 0.57 a	1.33 ± 0.12 b	1.28 ± 0.20 b	1.28 ± 0.19 c	139.20 ± 9.19 e	143.17 ± 20.01 d	142.44 ± 7.88 d	0.10 ± 0.01 a	0.07 ± 0.01 c	0.05 ± 0.01 b
	Na180	6.41 ± 0.28 b	4.62 ± 0.18 c	3.22 ± 1.01 c	0.87 ± 0.30 c	0.77 ± 0.12 c	0.60 ± 0.05 d	169.71 ± 17.24 c	181.90 ± 16.13 bc	166.82 ± 5.35 c	0.07 ± 0.00 bc	0.03 ± 0.01 e	0.03 ± 0.00 c

Note: Control, Na120, and Na180 represent Hoagland nutrient solution treatments containing 0, 120, and 180 mM NaCl, respectively, while 3 d, 6 d, and 9 d indicate the timepoints of 3, 6, and 9 days after NaCl treatment, respectively. ‘S37’ and ‘S39’ represent salt-sensitive and salt-tolerant processing tomato genotypes, respectively. Abbreviations: *A*_net_: Net photosynthetic rate; *C*_i_: Intercellular CO_2_ concentration; *g*_s_: Stomatal conductance; *E*: Transpiration rate. The data in the table represent the mean ± standard deviation. Each treatment had three biological replicates. Statistical significance was determined by one-way ANOVA followed by Duncan’s multiple. A *p*-value < 0.05 was considered statistically significant, and significant differences are indicated by different letters.

## Data Availability

The original contributions presented in the study are included in the article, and further inquiries can be directed to the corresponding author.

## Supplementary Materials

The following supporting information can be downloaded at https://www.mdpi.com/article/10.3390/plants15101450/s1, Table S1: Equations and definitions of JIPTest parameters obtained from chlorophyll a fluorescence emission (OJIP) analyses. Table S2: Analysis of significant differences in radar plots.
